# Potassium application enhances vegetative and reproductive yield of *Zygopetalum maculatum* and reduces post-flowering K depletion from storage organs of the orchid

**DOI:** 10.1038/s41598-025-89452-9

**Published:** 2025-03-29

**Authors:** Siddhartha Sankar Biswas, Suman Natta, N. S. Kalaivanan, H. Chandan Gowda, L. C. De, S. P. Das

**Affiliations:** ICAR-National Research Centre for Orchids, Pakyong, Sikkim India

**Keywords:** Orchid, *Zygopetalum maculatum*, Potassium, K content, K dynamics, Biological techniques, Physiology, Plant sciences

## Abstract

The orchid cultivation is a significant sector in floriculture industry, and *Zygopetalum maculatum* is one of the most important orchid species of this industry due to its captivating fragrance and aesthetic appeal. Orchids, being epiphytic, are typically grown in soilless media, which lack essential macronutrients like nitrogen (N), phosphorus (P) and potassium (K), crucial for overall plant growth. Literature cited suggest several studies on combined effect of NPK on orchids, however, the studies on the impact of sole K application on morphological traits, and flower yield of *Zygopetalum maculatum* orchid have not been cited. Hence, this study was designed to explore the impact of K supplementation on morphological traits, floral yields, K uptake by the flowers, K dynamics in plant parts, and vase life of *Zygopetalum maculatum* flowers. The experiment was laid out in completely randomized design with six treatments of K application (i.e. T_1_ = No K in fertigation solution (K_0_), T_2_ = 10 mg K per L fertigation solution (K_10_), T_3_ = 25 mg K per L fertigation solution (K_25_), T_4_ = 50 mg K per L fertigation solution (K_50_), T_5_ = 75 mg K per L fertigation solution (K_75_) and T_6_ = 100 mg K per L fertigation solution (K_100_)), each treatment was replicated four times. The plants under the experimentation were treated with the nutrient solution weekly once. Results showed that K application enhanced water-extractable K content and dehydrogenase activity in the potting media. Morphological parameters such as bulb size, leaf number were significantly increased under the K_100_ treatment. Floral yields, including spike length, floret number per spike, floret dimension, and flower biomass, were also substantially higher with K supplementation. The K_100_ treatment produced 167% higher number of flower spike per plant with 44.4% higher number of significantly bigger sized florets per spike over K_0_ treatment. K content in leaves, bulbs, and roots significantly increased with K application. Flowering induced K reduction from back bulbs, leaves and roots. The post-flowering K reduction from different plant parts was minimized by K_100_ treatment. Partial regression analysis showed one unit K uptake by flowers caused, ~ 0.227, 0.564 and 0.317 unit K reduction from leaf, back bulb, and roots, respectively. Moreover, flowers from the K_100_ treatment exhibited an extended vase life compared to other treatments. Thus, it can be recommended that, 100 mg K L^−1^ fertigation solution should be applied weekly to sustainably improve *Zygopetalum maculatum* yields.

## Introduction

During the early nineteenth century, orchids were being cultivated by hobbyist growers only. But, at present this crop has gained huge popularity and attained the position of a multimillion-dollar industry. Orchids, renowned for their elegant appearance^1^ and extended longevity, have gained significant popularity among floriculture enthusiasts and consumers^2^. These flowers are prized for their fascinating beauty, colors, and unique shapes^3,4^. Their popularity is reflected by their substantial presence in the market, accounting for over 10% of the international trade of potted plants, with a steady annual growth of 3.0% in the global cut flowers market^5^. *Zygopetalum maculatum* orchid is known for its highly attractive and fragrant flowers, making it a valuable asset in the orchid industry^6,7^. This species has gained popularity for its commercial potential as both cut flower and potted orchid^8^. The market demand for *Zygopetalum maculatum* flowers largely from its aesthetic appeal and fragrance, making it a prominent variety in floral trade. Furthermore, its scent profile has triggered research interest, particularly in the context of volatile compounds and their role in pollination mechanisms^9–11^. The growing importance of *Zygopetalum maculatum* in the orchid industry highlights the need to further explore its commercial cultivation and floral characteristics^12^.

Epiphytic orchids like *Zygopetalum maculatum* have adaptive mechanisms to thrive in moisture and nutrient limited environments^13^. Like, (i) orchid roots have velamen tissue, a spongy structure, it efficiently absorbs and retains water and nutrient solutions, minimize loss and ensure their subsequent effective utilization within the plant^14^, (ii) In orchids, water and nutrients are rapidly taken up and stored in various storage organs, which are later supplied to the growing plant parts as per requirement^13^. In case of *Zygopetalum*, pseudobulbs and roots act as storage organs^15^, and iii) Orchids employ strategies like Crassulacean Acid Metabolism (CAM) and develop highly impermeable cuticles on their leaves to mitigate water loss^16^. Among these adaptation mechanisms, the ability of orchids to efficiently absorb, store, recycle, and utilize nutrient solutions is crucial for their survival and proliferation in resource-limited conditions^15^. However, studies are rare on the storage of specific nutrient like K, and its redistribution in different orchid plant parts at their various growth stages.

Potassium (K) is the second most abundant essential nutrient in plant. It is vital for plant functionality^13^. In plants, K regulates a wide range of physiological processes, including strengthening plant structure, supporting overall growth^16^, promoting the movement of photosynthates, enhancing resistance to pests and diseases, and improving water management and drought tolerance^17^. At the cellular level, it plays a crucial role in maintaining ion homeostasis^18^, facilitate protein synthesis, osmoregulation, enzyme activation, and regulating membrane potential and charge balance in plant cells^19^. It also plays vital role in metabolic processes^20^, and photosynthesis^21^. and regulates the movement of nutrients, metabolites, and water within plants, influence xylem-phloem water transport and wood formation^22^.

Crops grown on soil-based growing media are abundantly supplied with K from different forms of soil K like water-soluble, exchangeable, non-exchangeable, and structural K^23^. But epiphytic orchids are typically grown in soilless, customized potting media^24^, designed to mimic their natural epiphytic conditions and provide optimal aeration and moisture at the root zone^25^. These media lack the natural soil backup for supplying K, thereby external K supplementation becomes crucial for efficient orchid cultivation. Extensive research on orchid nutrition has largely focused on the combined effects of nitrogen (N), phosphorus (P), and K application^13^. However, the specific impact of K application on orchid vegetative growths, flower yield, and vase life of cut flowers has been underexplored. Furthermore, the K content and dynamics across various orchid plant parts during different growth stages remained unexplored.

It was hypothesized that the flowering process and flowering-induced K uptake could deplete K content from the storage organs of the orchid, and K supplementation could enhance orchid vegetative and floral yields sustainably by minimizing K content reduction of the storage organs. To address these hypotheses, the study aimed to (i) Assess the impact of K application on morphological traits, floral yields, and K uptake by *Zygopetalum* flowers; (ii) Analyze K content and its dynamics in different plant parts at various growth stages of the orchid; and (iii) Evaluate the effect of K fertilization on the vase life of *Zygopetalum* cut flowers.

## Materials and methods

### Details of the pot experiment

#### Experimental site condition and planting materials

The experiment was conducted from the first week of April 2019 to the last week of May 2022 at Soil Science unit of the ICAR-National Research Centre for Orchids (ICAR-NRCO), Pakyong, Sikkim, India. Being a central government institute (dedicated exclusively to orchid related research), the institute has permission to work on any kind of orchid in India, and to work on *Zygopetalum maculatum* orchid proper permission was taken from the competent authority of the institute. The institute is the active germplasm site for orchids in India and houses Live specimens of *Zygopetalum maculatum* orchids (website: https://nrco.icar.gov.in/). These orchids have been identified, tissue cultured and conserved on a large scale by the institute. The orchid seeds were cultured with Gamborg B-5 basal medium supplemented with 25 g L^−1^ sucrose, 8 g L^−1^ Agar and 2.0 g L^−1^ charcoal. For the experiment, *Zygopetalum maculatum* orchids were obtained from the tissue culture section of the institute.

#### Polyhouse and environmental control

The orchids were cultivated in polyhouses at ICAR-NRCO, as per the method outlined by Biswas et al.^15^. The polyhouse under the experimentation was covered from the top with green coloured, 50% shade nets to provide filtered light to the orchids. The polyhouse (10 m × 5 m) was situated at 1390 m above mean sea level (AMSL), oriented in north to south direction with 3.66 m center height and 2.44 m side height. During the experimental period, day length in summer ranged from 11 to 13 h and from 8 to 10 h during winter, with light intensity from 460.5 to 555 µmol m⁻^2^ s⁻^1^ Photosynthetic Photon Flux Density. The polyhouse temperatures fluctuated between ~ 2 and 30 °C. The humidity levels during the experimentation were maintained between 60 and 75%.

#### Potting

**Fig. 1:**
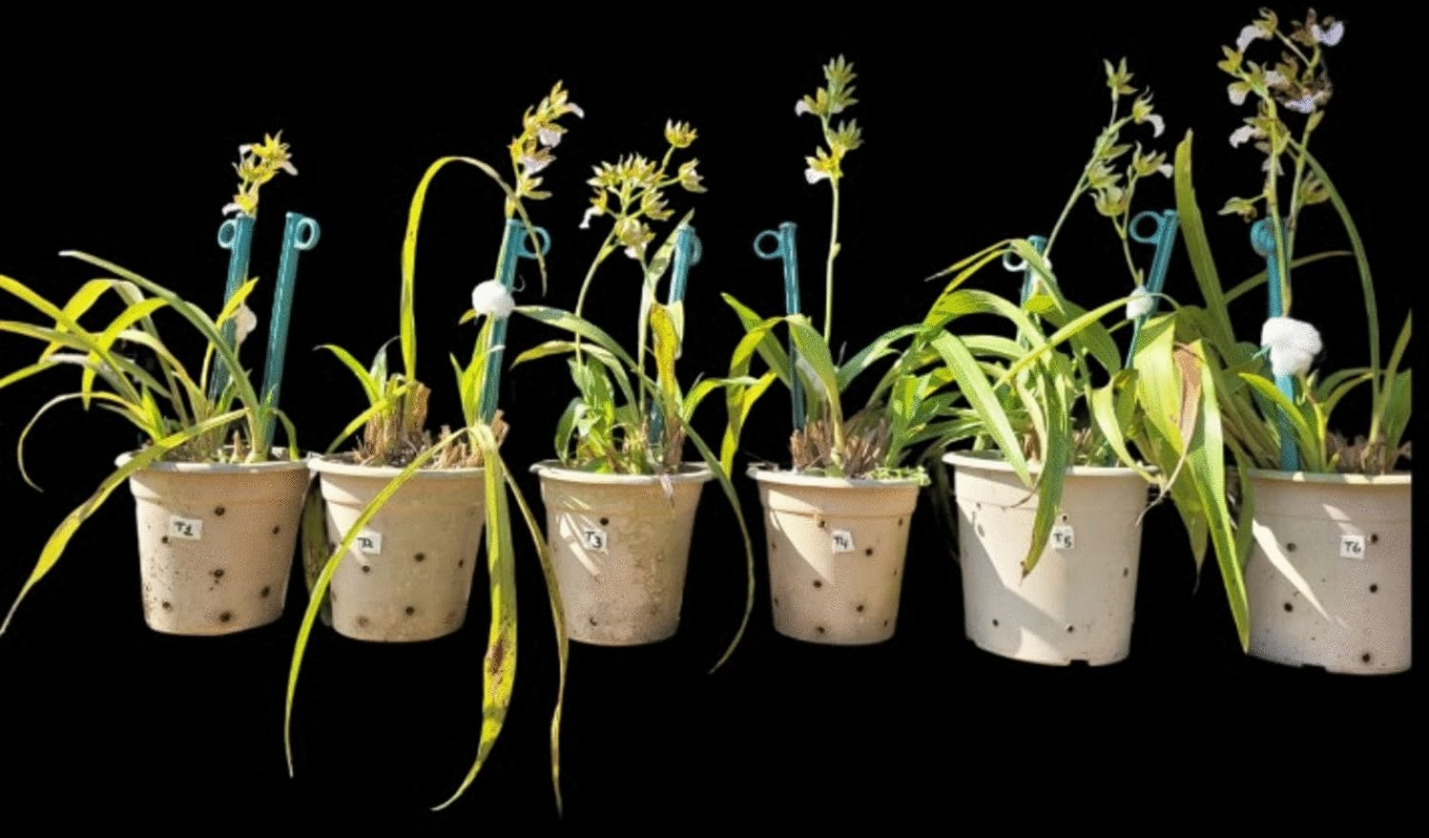
*Zygopetalum maculatum* plants under different treatments (T_1_ to T_6_ from left to right). T_1_ = Distilled water (control = K_0_), T_2_ = 10 mg L^−1^ K (K_10_), T_3_ = 25 mg L^−1^ K (K_25_), T_4_ = 50 mg L^−1^ K (K_50_), T_5_ = 75 mg L^−1^ K (K_75_) and T_6_: 100 mg L^−1^ K (K_100_).

The potting medium was prepared by 1:1:1:1 volumetric mixture of leaf mould, brick pieces, cocopeat, and coco chips. In April 2019, mature *Zygopetalum maculatum* plants were potted in 12 L plastic pots. Each plastic pot was provided with at least twelve 2 mm holes to ensure adequate root aeration. The potted plants were placed on tables elevated ~ 90 cm above ground level (Fig. 1).

#### Experimental details

The experiment comprised six treatments, each treatment was replicated four times. Each replication included two mature *Zygopetalum maculatum* plants. The treatments were, T_1_: Control (No external application of K, K_0_), T_2_: application of K @ 10 mg L⁻^1^ fertigation solution (K_10_), T_3_: application of K @ 25 mg L⁻^1^ fertigation solution (K_25_), T_4_: application of K @ 50 mg L⁻^1^ fertigation solution (K_50_), T_5_: application of K @ 75 mg L⁻^1^ fertigation solution (K_75_), and T_6_: application of K @ 100 mg L⁻^1^ fertigation solution (K_100_). The K was supplied through potassium sulphate (K₂SO₄) in the fertigation solutions, ensuring complete wetting of both potting medium and foliage during each application. Each pot was fertigated weekly once (from 10 to 11 AM) and received 300 mL of the respective fertilizer solution. The fertigation solutions for all treatments contained 100 mg L⁻^1^ N, 50 mg L⁻^1^ P, 25 mg L⁻^1^ Ca, 10 mg L⁻^1^ Mg, 10 mg L⁻^1^ Fe, 4 mg L⁻^1^ Cu, 10 mg L⁻^1^ Mn, 5 mg L⁻^1^ Zn, 2 mg L⁻^1^ Ni, 2 mg L⁻^1^ B, and 2 mg L⁻^1^ Mo. The plants bloomed in November 2021 and November 2022.

#### Preparation of fertilizer solution

The following chemicals i.e. ammonium sulfate [(NH₄)₂SO₄], ammonium dihydrogen phosphate [NH₄H₂PO₄], Calcium sulfate [CaSO₄], magnesium sulfate [MgSO₄], ferrous sulfate heptahydrate [FeSO₄·7H₂O], copper sulfate [CuSO₄], manganese sulfate heptahydrate [MnSO₄·7H₂O], zinc sulfate heptahydrate [ZnSO₄·7H₂O], nickel chloride hexahydrate [NiCl₂·6H₂O], boric acid [H₃BO₃], and ammonium molybdate [(NH₄)₆Mo₇O₂₄] were used as sources of N, P, Ca, Mg, Fe, Cu, Mn, Zn, Ni, B, Mo respectively. These analytical-grade chemicals were sourced from Merck Life Science Private Limited, Mumbai, India. Stock solutions were prepared from each salt in such a way that 1 mL of the stock solution contained the desired amount of each nutrient for 1 L of working solution. The electrical conductivity (EC) of the working solution varied 450 µS cm^−1^ to 481 µS cm^−1^, with EC of T_1_ solution 450 µS cm^−1^, T_2_ solution 454 µS cm^−1^, T_3_ solution 460 µS cm^−1^, T_4_ solution 468 µS cm^−1^, T_5_ solution 475 µS cm^−1^ and T_6_ solution 481 µS cm^−1^.

#### Record of observations

Morphological parameters were recorded during November 2022, those included bulb length, bulb width, leaf number, leaf length, and leaf width. Floral characteristics i.e. spike length, spike stem width, peduncle length, pedicel length, number of florets per spike, floret length, and floret width were also recorded (Supplementary Figure S1). For measuring length and width of any plant part digital vernier caliper and measuring tape were used. For K content analysis, samples from distinct plant parts (i. e. leaves, roots, back bulbs (BBs), and new bulbs (NBs)) were collected approximately one month before flower initiation and immediately after flower harvesting.

### Assessment of the initial properties of the potting media

To assess the initial chemical properties of the potting media, the representative potting mixture was collected, air-dried under ambient conditions, and subsequently oven-dried at ~ 68 °C for ~ 72 h (until it gained constant weight). The dried media were then crushed using a pestle and mortar and homogenized to ensure uniformity. The pH of the media was determined using a sample-to-water ratio of 1:10^15^. As the potting media contains coco chips and coco peat, in this case, a 1:10 sample-to-water ratio was used to have sufficient aliquot for measuring the pH of the potting media. Total N content was measured using the micro-Kjeldahl method. For the determination of P, K, calcium (Ca), and sodium (Na) content, the media samples were digested in a di-acid mixture (HNO₃ and HClO₄ :: 9:4). P content in the digested samples was estimated spectrophotometrically, using the vanadomolybdo phosphate yellow color method, while Na (at 589 nm wavelength with orange-yellow flame), K (at 766 nm wavelength with violet or lilac (pink) flame), and Ca (at 622 nm wavelength with orange-red flame) contents were determined using flame photometry^26^. Dehydrogenase activity, an important indicator of oxidative activity by microbial communities in the media, was evaluated according to the protocol by Camiña et al.^27^. The initial analysis revealed that the potting media had a near-neutral pH (6.93). Elemental analysis showed presence of 0.57% N, 0.21% P, 0.40% K, 0.36% Ca, and 0.39% Na in the potting media. The potting media also exhibited 3.12 μg TPF g⁻^1^ potting media h⁻^1^ dehydrogenase activity.

### Plant sample collection and processing

**Fig. 2:**
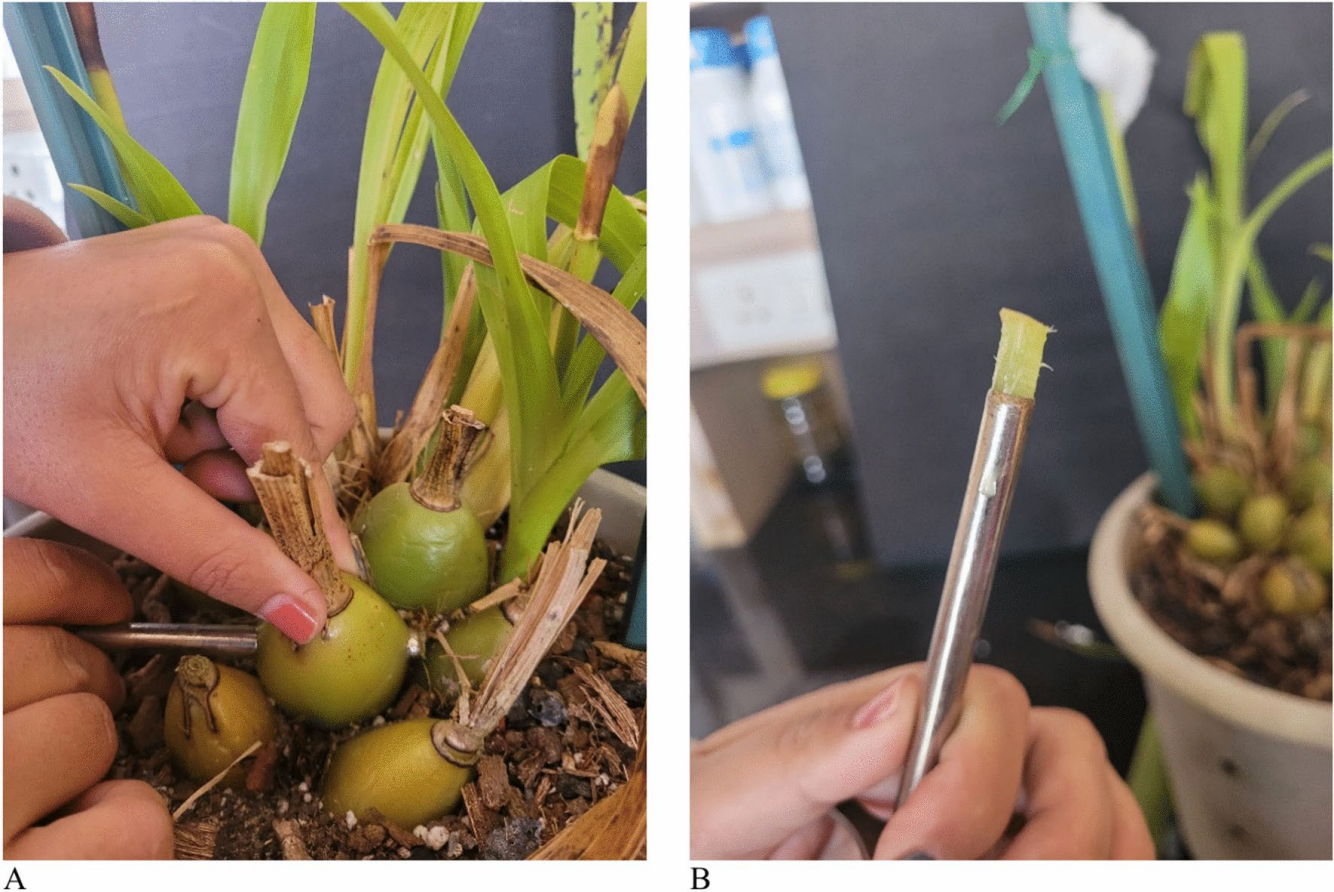
*Zygopetalum maculatum* bulb sample collection method (**A**) and, collected sample (**B**).

A non-destructive sampling of the plant samples of this perennial crop was conducted both before and after flowering to analyze K content in various crucial plant parts, like leaves, BBs, NBs, and roots. From each pot, nearly 3–5 g healthy root samples and a fully opened but recently matured leaf sample was also collected. A 4 mm cork borer was used to collect BB and NB samples. At least three samples from each pot were collected by horizontally inserting the cork borer through the bulbs from one end to the other. The material inside the cork borer was used for processing and subsequent analysis (Fig. 2)^15^. The fresh weight of all the collected samples was recorded, followed by controlled air drying under shade. Subsequently, the samples were oven-dried at ~ 68 °C until they reached a constant weight. The oven-dried samples were then digested using a di-acid mixture (HNO₃ : HClO₄ :: 9:4). A flame photometer was used to measure the K content in the digested plant samples.

### Assessment of moisture content in plant samples

Plant samples were weighted immediately after collection and the fresh weight of the samples were recorded. Samples were then air-dried for 24 h in shade, subsequently oven dried at ~ 68 °C until a constant weight was achieved. Thereafter, the dry weight of the samples were recorded. Moisture content was calculated using the equation below:

$${\text{Moisture}}\; {\text{content}} \left( \% \right) \, = \frac{{\left( {X - Y} \right)}}{X} \times 100$$

where X is the sample’s fresh weight and Y is the same sample’s dry weight.

### Monitoring potting media pH

The potting media pH was recorded at 0, 30, 90, 150, 210, 290, 370, 450, and 650 days after potting the plants. During each sampling, ~ 20–30 g of potting media was collected from each pot and mixed to form composite samples for each replication. Five grams of each composite sample was placed in a 100 mL beaker, and 50 mL of distilled water was added with a dilution of 1:10. The mixture was stirred vigorously for 5 min, and the pH was recorded immediately by microprocessor pH/mV/Temp. meter of reliable lab equipment (model number RLE—231 K). After pH measurement, the remaining potting media was returned to its respective pot.

### Water extractable K (WE-K) content in the potting media

For water extractable K (WE-K) content analysis, 5 g composite potting media sample from each replication was taken in a 150 mL conical flask, and 50 mL double distilled water was added to it. The mixture was shaken for 30 min then filtered the liquid extract. The K content in the extract was measured by using flame photometry^28^.

### Assessment of vase life of the cut flowers

**Fig. 3:**
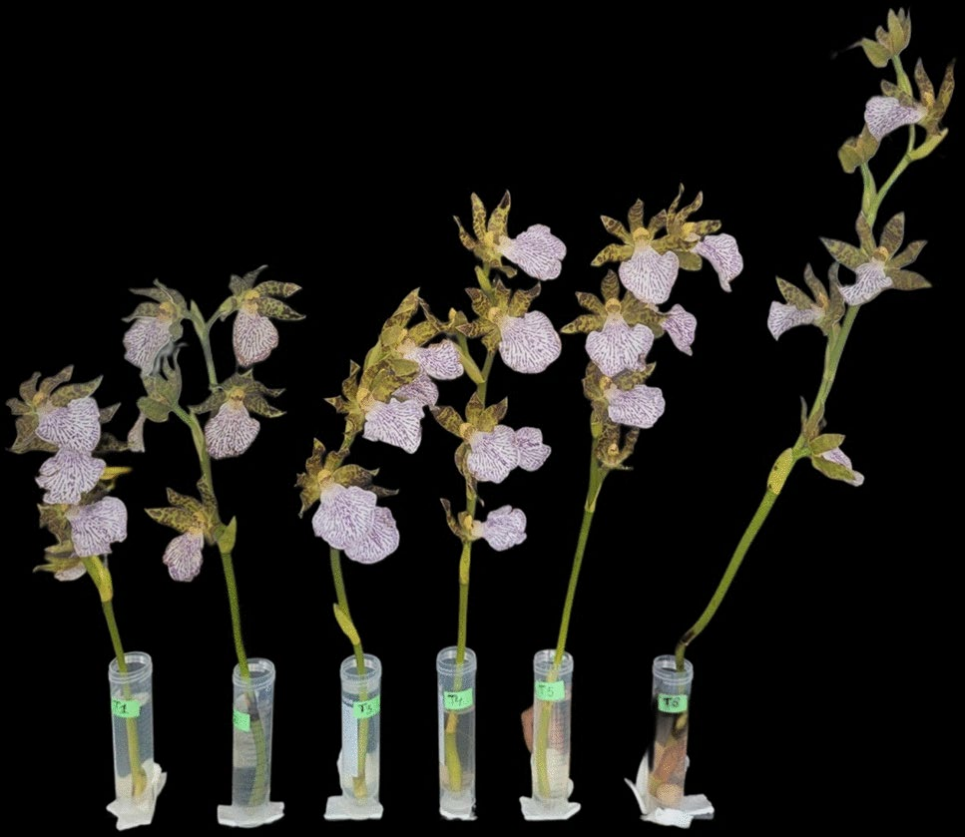
*Zygopetalum maculatum* flowers under vase life study.

The experimental procedure outlined by Biswas et al.^29^ was followed to assess the vase life of *Zygopetalum maculatum* cut flowers. For the study,18 spikes (three spikes per treatment across three replications) were harvested, leaving one cm base of each spike. The spikes were harvested when the topmost flower bud had just begun to open. Uniform plastic tubes (transparent, cylindrical, 50 mL) were used as flower vases (Fig. 3). Each spike was placed in 40 mL of slightly acidified distilled water (pH adjusted to 6 using citric acid), supplemented with 1 mL of streptomycin (100 mg L⁻^1^) as a biocide. The vase solution was replenished to 40 mL at 3 days intervals. The vase life of each flower spike was monitored until the detachment of the first floret from the spike^29^. The experiment was done in room condition; with ~ 10 h daylight, light level varied from 20.8 to 22.2 µmol m^−2^ s^−1^ Photosynthetic Photon Flux Density, the room temperature during the study ranged from 17.8 to 20 °C and relative humidity varied from ~ 60–70%.

### Experimental design and statistical analysis

The pot experiment was conducted with a Completely Randomized Design (CRD). The analysis of data from the experiments was conducted using the OPSTAT online portal (http://14.139.232.166/opstat/) under one factor CRD^30^. Analysis of variance (ANOVA) was performed, and the least significant difference (LSD) among treatment means at *p* ≤ 0.05 was calculated^31^ (Supplementary file 1). To compare the K content in different plant parts before and after flowering, paired t-tests were conducted using Microsoft Excel. Partial regression analysis was performed using R statistical software (version 4.3.1, 2023-06-16 ucrt)^15,32^, with K uptake in flowers as the independent variable and flowering-induced K content reduction in different plant parts as dependent variables.

## Result

### pH of the potting media

The initial pH of the potting media was neutral and statistically similar under all the treatments. Potting media pH started declining from neutral to acidic pH with time in all the treatments (Table 1). The fall of pH was more pronounced in K untreated control treatment (K0) and under K_100_ treatment the fall of pH was minimal. From 90 days onward significant pH difference was observed under K_0_ and K_100_ treatments. In K_100_ treatment the potting media pH was 3.51%, 4.01%, 4.33%, 5.81%, 6.83%, 8.44%, and 10.1% higher over the potting media pH in K_0_ treatment at 90, 150, 210, 290, 370, 450, and 650 days after potting. During 650 days experimentation, under K_0_ treatment the fall of pH was ~ 207% higher over that of under K_100_ treatment.

**Table 1:** The pH dynamics in potting media over a period of 650 days of *Zygopetalum maculatum* cultivation.

Treatment	pH of the potting media (Days after potting plants)								
	0	30	90	150	210	290	370	450	650
T_1_	6.93a	7.06a	6.83b	6.74b	6.69b	6.54b	6.44b	6.28c	6.07c
T_2_	6.92a	7.04a	6.82b	6.77b	6.71b	6.59b	6.47b	6.31c	6.12c
T_3_	6.94a	7.05a	6.81b	6.79b	6.75b	6.63b	6.52b	6.40bc	6.21bc
T_4_	6.91a	7.03a	6.84b	6.78b	6.76b	6.64b	6.54b	6.43bc	6.29bc
T_5_	6.95a	7.09a	6.87b	6.81b	6.78b	6.71ab	6.66ab	6.57ab	6.38b
T_6_	6.96a	7.11a	7.07a	7.01a	6.98a	6.92a	6.88a	6.81a	6.72a

Means with statistically significant differences (as per LSD, *P* < 0.05) are denoted by distinct lowercase letters within treatments. T_1_ = Distilled water (control = K_0_), T_2_ = 10 mg L^−1^ K (K_10_), T_3_ = 25 mg L^−1^ K (K_25_), T_4_ = 50 mg L^−1^ K (K_50_), T_5_ = 75 mg L^−1^ K (K_75_) and T_6_: 100 mg L^−1^ K (K_100_), Potting media consisted 1:1:1:1 volumetric mixture of leaf mould, brick pieces, cocopeat, and coco chips.

### Water extractable K (WE-K) and dehydrogenase activity of the potting media

**Fig. 4:**
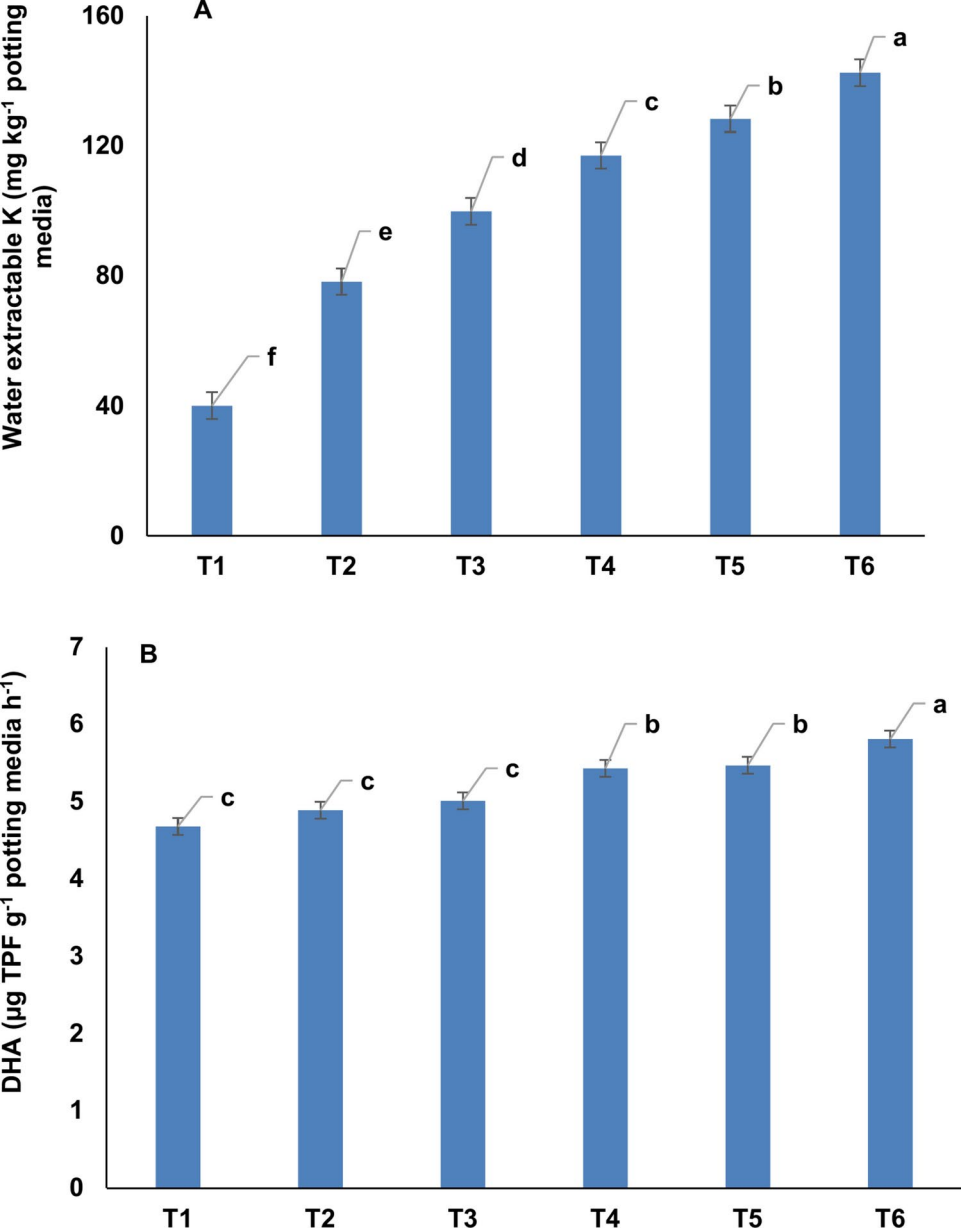
Post-flowering water extractable K content (**A**) and dehydrogenase activity (**B**) in *Zygopetalum maculatum* orchids potting media. Means with statistically significant differences (as per LSD, *P* < 0.05) are denoted by distinct lowercase letters within treatments. T_1_ = Distilled water (control = K_0_), T_2_ = 10 mg L^−1^ K (K_10_), T_3_ = 25 mg L^−1^ K (K_25_), T_4_ = 50 mg L^−1^ K (K_50_), T_5_ = 75 mg L^−1^ K (K_75_) and T_6_: 100 mg L^−1^ K (K_100_).

Post-flowering WE-K content in the potting media significantly increased under K treatments. The K_10_, K_25_, K_50_, K_75,_ and K_100_ treatments showed ~ 95.3%, 149%, 192%, 220%, and 256% higher WE-K as compared to K_0_ treatment (Fig. 4A). At this stage, the dehydrogenase activity of the potting media also increased significantly under K_50_, K_75,_ and K_100_ treatments over other treatments (Fig. 4B). The K_50_, K_75,_ and K_100_ treatments demonstrated 16.0%, 16.9%, and 24.1% higher dehydrogenase activity in the potting media over K_0_ treatment.

### Vegetative and floral yield of the orchid

**Fig. 5:**
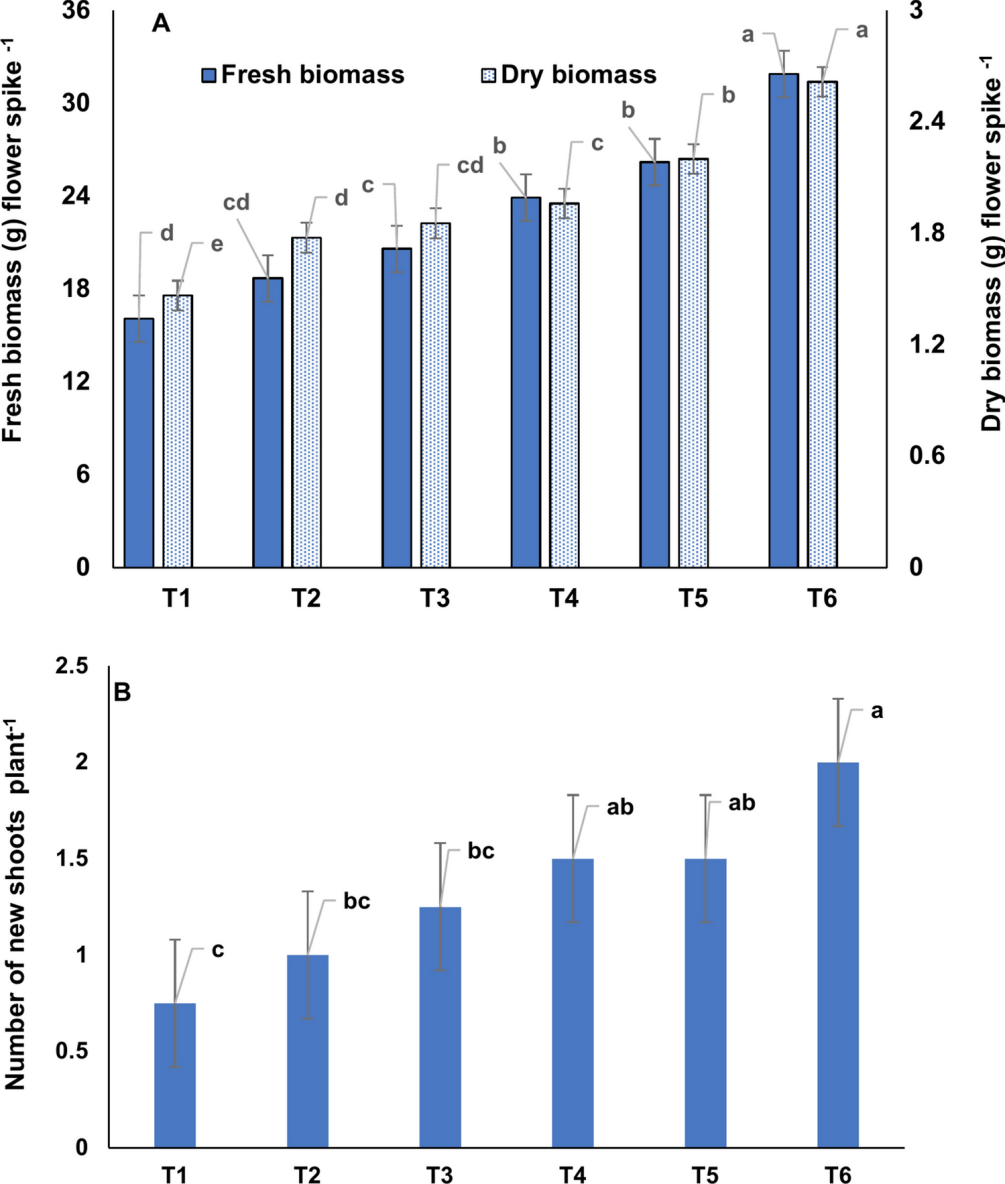
Fresh and dry biomass of *Zygopetalum maculatum* flowers (A), and emergence of new shoots per plant under different levels of K application. Means with statistically significant differences (as per LSD, *P* < 0.05) are denoted by distinct lowercase letters within treatments. T_1_ = Distilled water (control = K_0_), T_2_ = 10 mg L^−1^ K (K_10_), T_3_ = 25 mg L^−1^ K (K_25_), T_4_ = 50 mg L^−1^ K (K_50_), T_5_ = 75 mg L^−1^ K (K_75_) and T_6_: 100 mg L^−1^ K (K_100_).

The vegetative growth of different plant parts of the orchid was also significantly higher under K_100_ treatment (Table 2). Under the K_100_ treatment, the bulb length, bulb width, number of leaves per plant, leaf length, and leaf width were ~ 28.9%, 24.9%, 38.5%, 39.5% and 28.4% higher over K_0_ treatment. Besides vegetative yields, floral yields also enhanced with K application. The K_100_ treatment produced ~ 167% higher number of flower spikes per plant, over K_0_ treatment (Table 3). Under this treatment the spike length as well as spike stem diameter was also enhanced by ~ 33.5% and 55.4% respectively, over K_0_ treatment. Along with spike dimensions, the florets number per spike as well as floret dimension also significantly enhanced under this treatment. The K_100_ treatment produced ~ 44.4% higher number of florets (with ~ 30.3% and 28.3% higher floret length and width) per spike over K_0_ treatment. In consequence, the fresh and dry biomass of the flower spikes significantly enhanced under K_100_ treatment. This treatment produced ~ 98.1% and 78.5% higher fresh and dry biomass respectively, over K_0_ treatment (Fig. 5A). After flowering new shoot initiation from the orchids determines the future performance of these perennial crops. It was observed that K application increased the rate of new shoot initiation from the plants (Fig. 5B). The K_100_ treatment produced maximum number of new shoots, and the number of new shoots from this treatment were ~ 167% higher over K_0_ treatment.

**Table 2:** Effect of K application on bulb and leaf characteristics of *Zygopetalum maculatum* plant at flowering stage.

Treatment	Bulb length (cm)	Bulb width (cm)	Number of leaves	Leaf length (cm)	Leaf width (cm)
T_1_	5.19c	4.69c	6.50e	38.0d	3.91c
T_2_	5.51bc	4.67c	6.75de	37.9d	3.99c
T_3_	5.59bc	5.10bc	7.25cd	42.3c	4.09c
T_4_	5.86b	5.17bc	7.50c	47.3b	4.25bc
T_5_	6.11ab	5.68ab	8.25b	50.73a	4.56b
T_6_	6.69a	5.86a	9.00a	53.0a	5.02a

Means with statistically significant differences (as per LSD, *P* < 0.05) are denoted by distinct lowercase letters within treatments. T_1_ = Distilled water (control = K_0_), T_2_ = 10 mg L^−1^ K (K_10_), T_3_ = 25 mg L^−1^ K (K_25_), T_4_ = 50 mg L^−1^ K (K_50_), T_5_ = 75 mg L^−1^ K (K_75_) and T_6_: 100 mg L^−1^ K (K_100_).

**Table 3:** Effect of K application on spike and floret characteristics of *Zygopetalum maculatum* plants.

Treatment	Number of spikes per plant	Spike length (cm)	Spike stem width (cm)	Peduncle length (cm)	Pedicle length (cm)	Number of florets per spike	Floret length (cm)	Floret width (cm)
T_1_	0.75b	53.1d	0.621d	28.2c	4.52c	4.50c	6.31c	5.52c
T_2_	1.00b	55.6cd	0.772c	31.4b	4.97c	5.00c	6.51c	6.11bc
T_3_	1.25b	60.2bc	0.799c	31.5b	5.56b	5.25bc	6.97bc	6.16b
T_4_	1.50ab	64.4b	0.851bc	34.3ab	5.79b	5.75b	7.24b	6.46b
T_5_	1.75ab	65.3b	0.886b	35.8a	6.01ab	5.50bc	7.31b	6.54ab
T_6_	2.00a	70.9a	0.965a	36.1a	6.28a	6.50a	8.22a	7.08a

Means with statistically significant differences (as per LSD, *P* < 0.05) are denoted by distinct lowercase letters within treatments. T_1_ = Distilled water (control = K_0_), T_2_ = 10 mg L^−1^ K (K_10_), T_3_ = 25 mg L^−1^ K (K_25_), T_4_ = 50 mg L^−1^ K (K_50_), T_5_ = 75 mg L^−1^ K (K_75_) and T_6_: 100 mg L^−1^ K (K_100_).

### Moisture content in different plant parts

Moisture content in leaves, BBs, NBs, roots, and flowers ranged from 83.4% to 92.5%, 85.5% to 90.1%, 86.1% to 90.8%, 75.2% to 84.9%, and 90.9% to 91.8% respectively (Table 4). The K application enhanced moisture content in different plant parts except the flowers. Under K_100_ treatment, the moisture contents in leaves, BBs, NBs and roots were enhanced by 10.9%, 5.38%, 5.46%, and 12.9% respectively, over K_0_ treatment.

**Table 4:** Moisture content in *Zygopetalum maculatum* plant parts under different levels of K application.

Treatment		Moisture content (%)			
	Leaf	Back bulb	New bulb	Flower	Root
T_1_	77.3c	85.2b	88.4a	91.8a	73.5b
T_2_	79.6bc	87.7ab	88.5a	92.3a	75.2b
T_3_	82.1ab	88.4ab	88.7a	92.5a	76.1b
T_4_	82.2ab	87.6ab	90.4a	92.9a	79.7ab
T_5_	85.7ab	90.3a	90.2a	92.1a	78.6ab
T_6_	88.4a	90.9a	90.6a	93.1a	82.3a

Means with statistically significant differences (as per LSD, *P* < 0.05) are denoted by distinct lowercase letters within treatments. T_1_ = Distilled water (control = K_0_), T_2_ = 10 mg L^−1^ K (K_10_), T_3_ = 25 mg L^−1^ K (K_25_), T_4_ = 50 mg L^−1^ K (K_50_), T_5_ = 75 mg L^−1^ K (K_75_) and T_6_: 100 mg L^−1^ K (K_100_).

### Pre and post-flowering K content in different plant parts

Before flowering, in leaves, BBs, NBs, and roots the K content has ranged from 1.16% to 1.72%, 0.932% to 1.49%, 0.512% to 0.779%, and 1.36% to 1.91% respectively (Table 5). The maximum level of K content in different plant parts were observed under K_100_ treatment while the minimum was observed under K_0_ treatment. Under K_100_ treatment the K content in leaves, BBs, NBs, and roots were ~ 48.3%, 59.9%, 52.2%, and 40.4% higher respectively, over K_0_ treatment.

**Table 5:** Pre and post-flowering K concentration in *Zygopetalum maculatum* plant parts.

Treatment	K content (%) in different plant parts							
	Pre-flowering				Post-flowering			
	Leaf	Back bulb	New bulb	Root	Leaf	Back bulb	New bulb	Root
T_1_	1.16e	0.932d	0.512d	1.36c	0.982e	0.634f	0.509d	0.974d
T_2_	1.31d	0.949d	0.561 cd	1.61b	1.12d	674e	0.572c	1.31c
T_3_	1.46c	1.14c	0.584c	1.72b	1.31c	0.793d	0.591c	1.42c
T_4_	1.56bc	1.15c	0.651b	1.82ab	1.47b	0.958c	0.655b	1.55b
T_5_	1.58b	1.29b	0.66b	1.88ab	1.50b	1.09b	0.676b	1.61ab
T_6_	1.72a	1.49a	0.779a	1.91a	1.65a	1.30a	0.834a	1.69a

Means with statistically significant differences (as per LSD, *P* < 0.05) are denoted by distinct lowercase letters within treatments. T_1_ = Distilled water (control = K_0_), T_2_ = 10 mg L^−1^ K (K_10_), T_3_ = 25 mg L^−1^ K (K_25_), T_4_ = 50 mg L^−1^ K (K_50_), T_5_ = 75 mg L^−1^ K (K_75_) and T_6_: 100 mg L^−1^ K (K_100_).

**Fig. 6:**
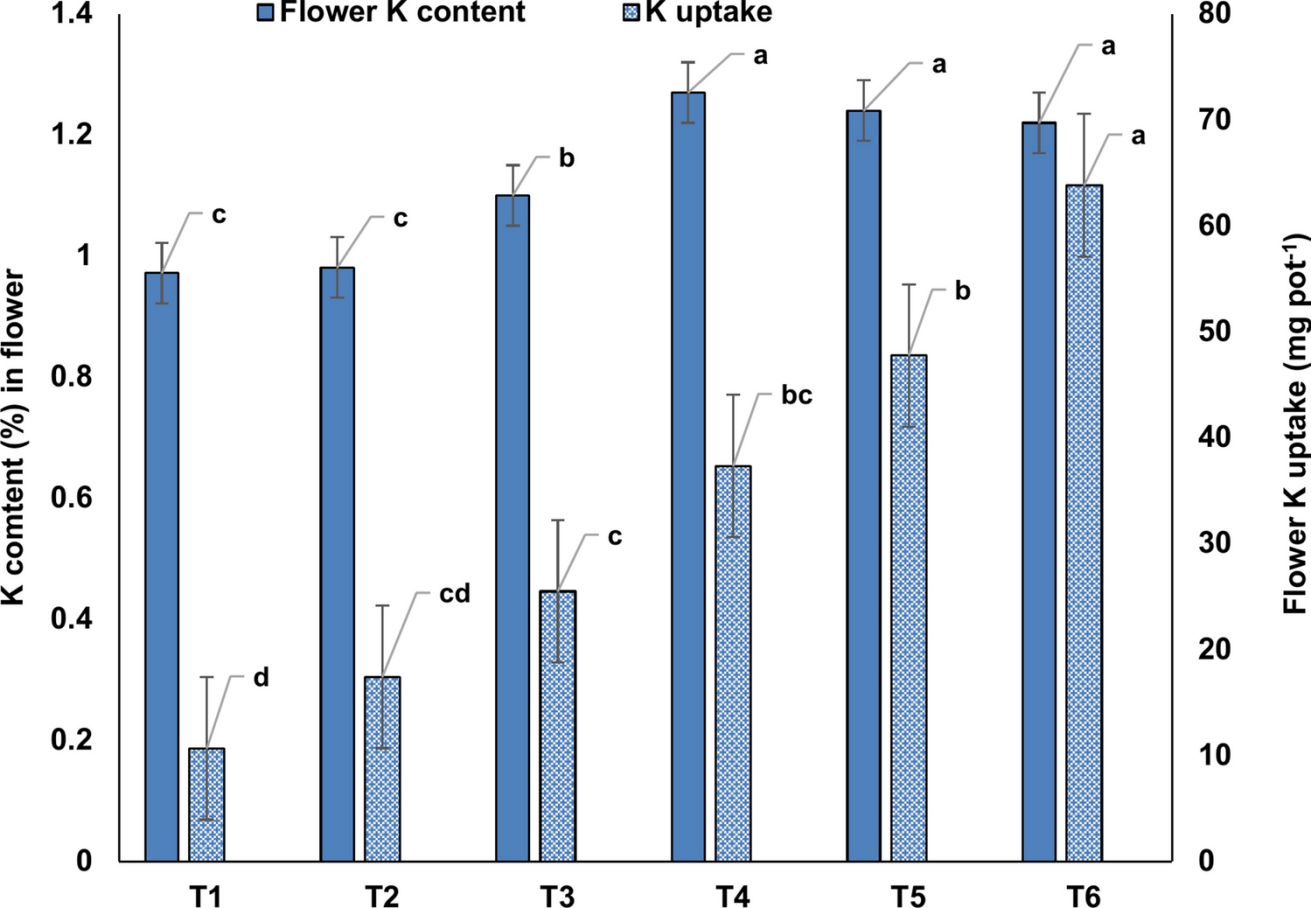
K content (%) and K uptake by *Zygopetalum maculatum* flowers. Means with statistically significant differences (as per LSD, *P* < 0.05) are denoted by distinct lowercase letters within treatments. T_1_ = Distilled water (control = K_0_), T_2_ = 10 mg L^−1^ K (K_10_), T_3_ = 25 mg L^−1^ K (K_25_), T_4_ = 50 mg L^−1^ K (K_50_), T_5_ = 75 mg L^−1^ K (K_75_) and T_6_: 100 mg L^−1^ K (K_100_).

The K content in flower ranged from 0.972% to 1.27% and the maximum flower K content was observed under K_50_ treatment (Fig. 6). The K content in flowers under K_50_, K_75,_ and K_100_ treatments were statistically at par. Under the K_100_ treatment, flower K content was 25.5% higher over K_0_ treatment. The K uptake was also maximum under the K_100_ treatment (Fig. 6). Under this treatment, the K uptake was ~ 498%, 266%, 150%, 71%, and 33.7% higher over those under K_0_, K_10_, K_25_, K_50_ and K_75_ treatments, respectively.

### K dynamics among different plant parts

**Fig. 7:**
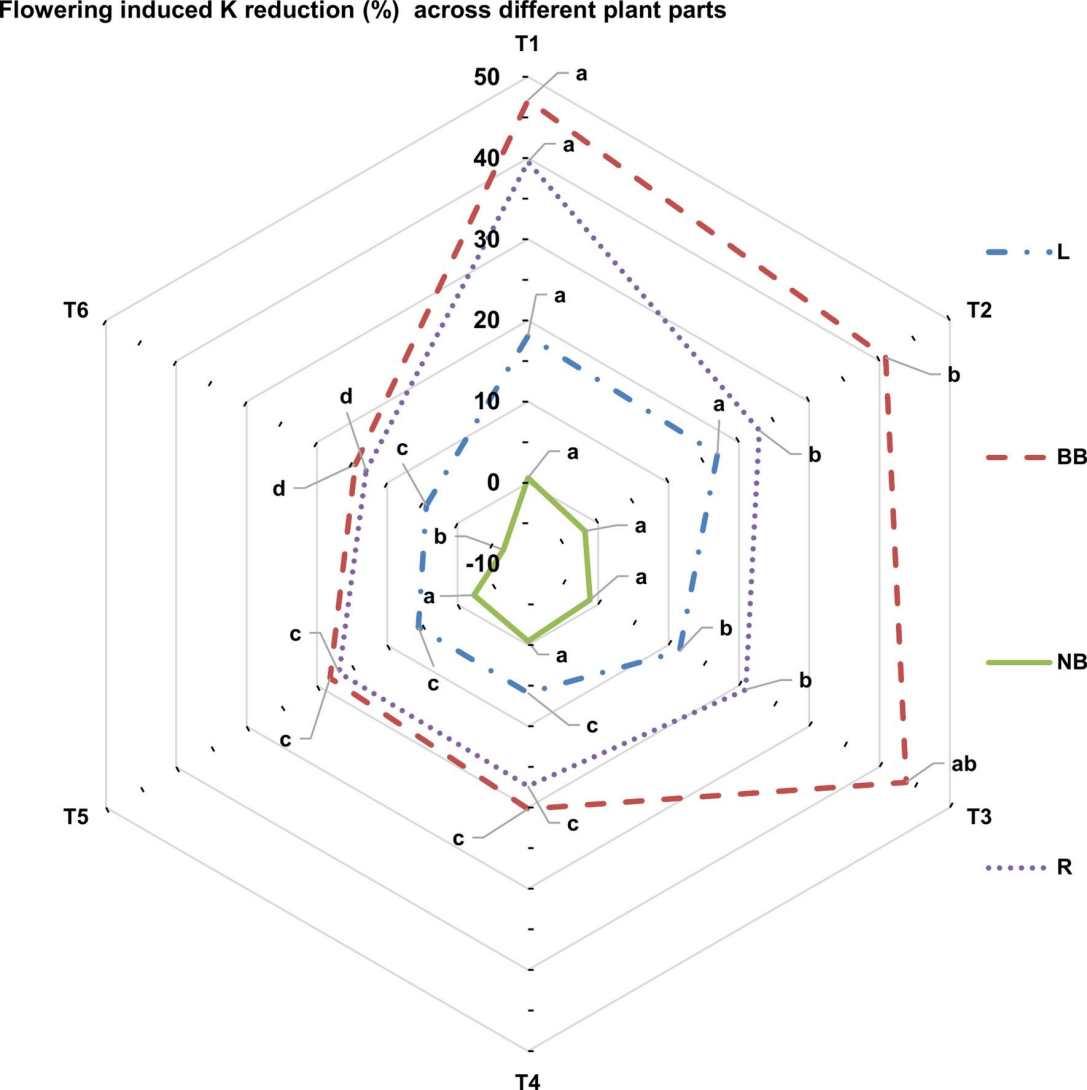
Post-flower K reduction (%) in different plant parts of *Zygopetalum maculatum* orchid. Means with statistically significant differences (as per LSD, P < 0.05) are denoted by distinct lowercase letters within treatments. T_1_ = Double distilled water (control = K_0_), T_2_ = 10 mg L^−1^ K (K_10_), T_3_ = 25 mg L^−1^ K (K_25_), T_4_ = 50 mg L^−1^ K (K_50_), T_5_ = 75 mg L^−1^ K (K_75_) and T_6_: 100 mg L^−1^ K (K_100_). L = Leaf, BB = Back bulb, NB = New bulb, and R = Root.

The flowering-induced K uptake decreased K content in different plant parts. The post-flowering K content in leaves, BBs, NBs, and roots ranged from 0.982% to 1.62%, 0.634% to 1.30%, 0.509% to 0.834%, and 0.974% to 1.69% respectively (Table 5). The Paired t test results proved that the post-flowering K content reduced significantly in all the plant parts except NBs (Table 6). Among the plant parts, the post-flowering K content reduction was maximum in BBs followed by root, leaves and NBs (Fig. 7). Under K_100_ treatment, the K uptake was ~ 498% higher over K_0_ treatment. Despite this extremely higher K uptake under K_100_ treatment, the flowering-induced K content reduction was ~ 308%, 224%, and 204% lower in leaf, BB, and roots respectively, compared to K_0_ treatment. Partial regression analysis among the data of post-flowering K content reduction in different plant parts and K uptake by flowers revealed that K content reduction in leaf and K uptake by flowers has partial regression coefficient of − 0.2273 and intercept value of 18.13 (Table 7). The partial regression coefficient and intercept values for the BBs were − 0.5644 and 49.77, respectively, and for roots, those were − 0.3174 and 32.505, respectively.

**Table 6:** Results from the Paired t-test between pre and post-flowering K concentration in different *Zygopetalum maculatum* plant parts.

Variable	Variance BF	Variance AF	Pearson Correlation	t-Statistic	t Critical two-tailed	*p*-value (Two-tailed)
Leaf	1.465	1.338	0.996	5.992	2.571	0.00186
Back bulb	1.159	0.908	0.979	9.218	2.571	0.00025
New Bulb	0.6245	0.6395	0.996	−1.7819	2.571	0.134887
Root	1.7166	1.4256	0.9979	12.966	2.571	0.0000486

*BF* before flowering, *AF* after flowering.

**Table 7:** Results from partial regression analysis among potassium uptake (KU) by flowers and K content at different plant parts of *Zygopetalum maculatum* orchids.

Variable	Intercept	Regression coefficient of KU
L	18.13	−0.2273
BB	49.77	−0.5644
NB	0.8961	−0.0902
R	32.505	−0.3174

L = K content reduction in leaf, BB =K content reduction in back bulb, NB = K content reduction in new bulb and R = K content reduction root.

### Vase life of the orchid cut flowers

**Fig. 8:**
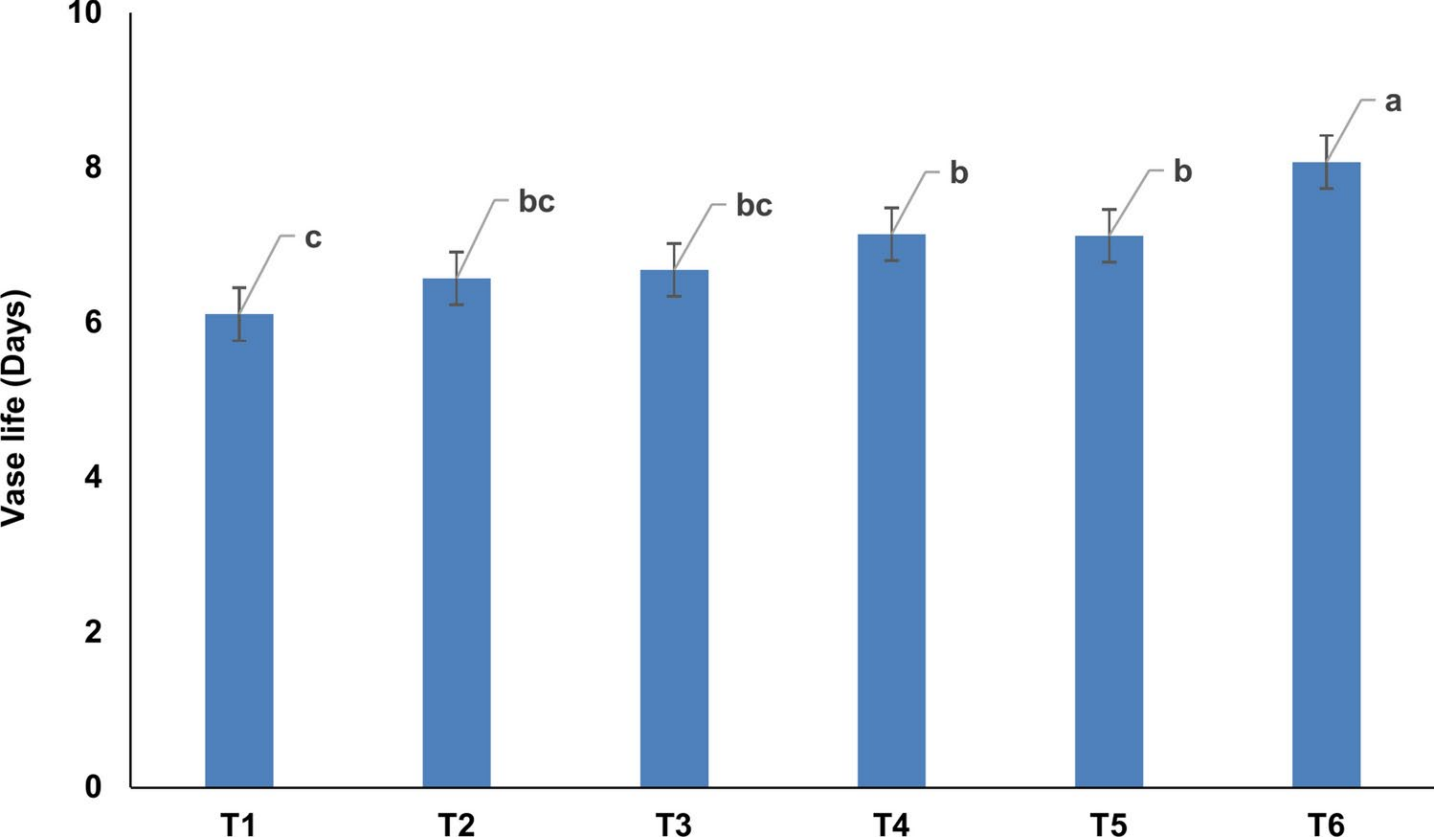
Vase life of *Zygopetalum maculatum* orchid cut flowers under different level of K application. Means with statistically significant differences (as per LSD, *P* < 0.05) are denoted by distinct lowercase letters within treatments. T_1_ = Double distilled water (control = K_0_), T_2_ = 10 mg L^−1^ K (K_10_), T_3_ = 25 mg L^−1^ K (K_25_), T_4_ = 50 mg L^−1^ K (K_50_), T_5_ = 75 mg L^−1^ K (K_75_) and T_6_: 100 mg L^−1^ K (K_100_).

The *Zygopetalum maculatum* flowers produced under externally supplied K exhibited enhanced vase life (Fig. 8). The flower spikes from K_100_ treatment showed ~ 32.1%, 22.8%, 20.8%, 13.0% and 13.3% extended vase life over the flowers from K_0_, K_10_, K_25_, K_50_, and K_75_ treatments, respectively.

## Discussion

The consistent preparation of the potting media for the cultivation of *Zygopetalum maculatum* orchid, included uniform mixture of cocopeat, coco chips, leaf mold, and brick pieces, ensured homogeneity and consistent pH across different treatments^33^. The organic matter of the potting medium began decomposing shortly after potting^15^, and released organic acids^15^. These process, combined with plant root exudates^34^, led to pH decline in all the treatments. However, the application of K (an alkaline metal), through various treatments diminished the potting medium pH reduction^35^, by providing a buffering effect. Conversely, in the control treatment (K_0_), which lacked an external source of K, there was a more significant pH decline. The higher application of K, administered as a fertilizer solution in the K_100_ treatment, likely increased the WE-K content in the potting medium significantly^36^.

Dehydrogenase activity serves as a key indicator of the oxidative activity of all the microbial communities within a growing medium, provides valuable insights into the overall microbial presence^37^. The observed increased vegetative growth under optimal K supply (Table 2) suggests enhanced health and vitality of the root system within the potting medium^38,39^. On the other hand, the decomposable organic matter in the potting medium, along with plant root exudates, provides essential food and energy sources for the microbial populations, thereby enhancing their activity and proliferation^40^, which significantly enhanced the dehydrogenase activity of the potting medium.

Availability of sufficient K under K_100_ treatment likely facilitated efficient water and nutrient uptake by the orchids, also K may have enhanced nutrient and carbohydrate translocation within the plants, which is crucial for optimal plant growth^22^. Consequently, the K_100_ treatment maximized vegetative growth and flower yield, which are primary interests for orchid growers. The continuous and ample K supply in the K_100_ treatment led to increased vegetative growth, enhanced floral quality, and improved floret and spike dimensions. Moreover, K facilitates water absorption and translocation within plants^22^, which elevated moisture content in leaf, roots, and BBs, thereby enhanced the freshness of various plant parts. This increased freshness improved nutrient and moisture absorption, thereby boosting both vegetative and reproductive yields in orchids. These findings are in agreement with those of El-Naggar et al.^41^, who reported positive effects of K foliar application on the vegetative and reproductive yields of Gladiolus hybrid, L. Cv. “Rose Supreme”. Similarly, Shah et al.^42^ documented that Dendrobium cv. Sonia 17 exhibited enhanced vegetative and flower yield in response to K application in conjunction with N. In the K_100_ treatment, the continuous supply of optimum quantity K, and its subsequent assimilation by plants led to increased K concentration in various plant parts^43^. In *Zygopetalum maculatum* orchids, from mature bulbs, flower spikes and new shoots emerge. With time at the base of the new shoots NBs starts forming, these NBs act as nutrient sinks. After flowering, the mature bulb may shed its leaves, and become defoliated BBs^15,25^. These BBs, and roots, serve as crucial reservoirs of moisture, nutrients, carbohydrates, and sugars, which are recycled during periods of scarcity to support new growth^13^. In *Zygopetalum* leaves, roots and bulbs undergo developmental senescence. This process of senescence facilitates nutrient recycling from leaves, BBs and roots which support the development of new organs and overall health of the orchid^15^. K is mobile within plants, it can be translocated from mature or dying plant parts to areas of active growth as needed^44^. The dynamic translocation of K from senescing leaves, BBs, and roots to new shoots and flowers reduced K concentrations in these organs. However, NBs, being active sinks, its K concentrations remained unaffected by the K demand of flowers, in fact with ample supply even its K concentration increased. Under the K_100_ treatment, the ample supply of K ensured its sufficient availability for flowers and newly growing shoots, which in consequence minimized the post-flowering K depletion from source organs such as leaves, BBs, and roots, under this treatment. In contrast, the K_0_ treatment, which lacked external K supply, forced the plant to rely solely on recyclable internal K reserves and the potting medium to support new growths. This led to a significant post-flowering K reduction in leaves, BBs, and roots.

Partial regression analysis revealed that for every unit of K uptake by the flower, the K content in leaf, BBs and roots decreased by ~ 0.227, 0.564, and − 0.317 units respectively^15,45^. The intercept coefficients highlighted the BBs as the most vulnerable to K content reduction due to flowering-induced K uptake, indicating a reduction of approximately 49.8 units at the level zero K uptake by flowers. At the same time, the reductions in K content in leaves and roots were less pronounced, with decreases of 18.13 and 32.505 units (Table 7), respectively^46^. Before flowering, plants undergo complex physiological processes to support reproductive growth, which involves shifts in metabolic activity^47^, and hormonal changes such as increased levels of auxins and cytokinins^48^. These processes facilitate the translocation of nutrients, including K, from older plant parts like BBs, leaves, and roots to actively growing or reproductive tissues such as flowers and new shoots^19,49^. This nutrient translocation declined K content in older plant parts just before flower initiation.

The study underscores the critical role of K in supporting the orchid growth and development, particularly during the flowering stage. The findings indicate that adequate external K supply is essential to mitigate the K depletion from older plant parts and to sustain overall plant health and productivity. The K_100_ treatment effectively maintained higher K levels in the plant parts, minimized post-flowering K content reductions in leaves, BBs, and roots. Conversely, in the K_0_ treatment, the lack of external K supply forced the plant to rely on its internal reserves, leading to significant K depletion in various parts of the plant.

The K_100_ treatment yielded flowers with significantly higher biomass. Thus, the flower spikes under this treatment had higher levels of stored carbohydrates. These stored carbohydrates are crucial for supporting metabolic activities of the cut flowers, that potentially extend their vase life by maintaining freshness for a longer period^15,29^. K also have a vital role in stomatal function and water uptake^22,50^. An optimal supply of K can improve stomatal functioning in flowers, that may enhance the uptake of vase solution. This increased vase solution uptake, combined with the higher carbohydrate content, maintained the turgidity of the flower spikes for longer periods. Consequently, the vase life of the flowers is extended under the K_100_ treatment.

## Conclusions

This study provides valuable insights into the role of K supplementation in optimizing the cultivation of *Zygopetalum maculatum* orchids. The study also demonstrate that K application significantly influences vegetative growth, floral yields, and post-harvest longevity of the orchid cut flowers. K uptake by flowers can cause K reduction from leaves, back bulbs, and roots, but not from young bulbs. The back bulbs are most vulnerable towards flowering induced K loss followed by roots and leaves. Adequate K supply can mitigate flowering induced K loss from different plant parts. These findings indicate K supplementation can sustainably enhance overall plant vigor of *Zygopetalum maculatum* orchids. The study concludes that supplementing 100 mg K L^−1^ fertigation solution applied weekly minimizes pH reduction and maintains near neutral pH of the potting media for enhancing quality yield of *Zygopetalum maculatum* orchids in a sustainable manner.

## Supplementary Information

Supplementary Information 1.

Supplementary Information 2.

## Data Availability

The datasets generated and/or analysed during the current study are available from the corresponding author on reasonable request.
