# Supplementary material for: Potassium application enhances vegetative and reproductive yield of Zygopetalum maculatum and reduces post-flowering K depletion from storage organs of the orchid
Source: Sci Rep. 2025 Mar 29;15:10907. doi: 10.1038/s41598-025-89452-9 (PMC11954905; doi:10.1038/s41598-025-89452-9)
Supplement: Supplementary file 1 — Supplementary Information 1. [file 41598_2025_89452_MOESM1_ESM.docx]

| 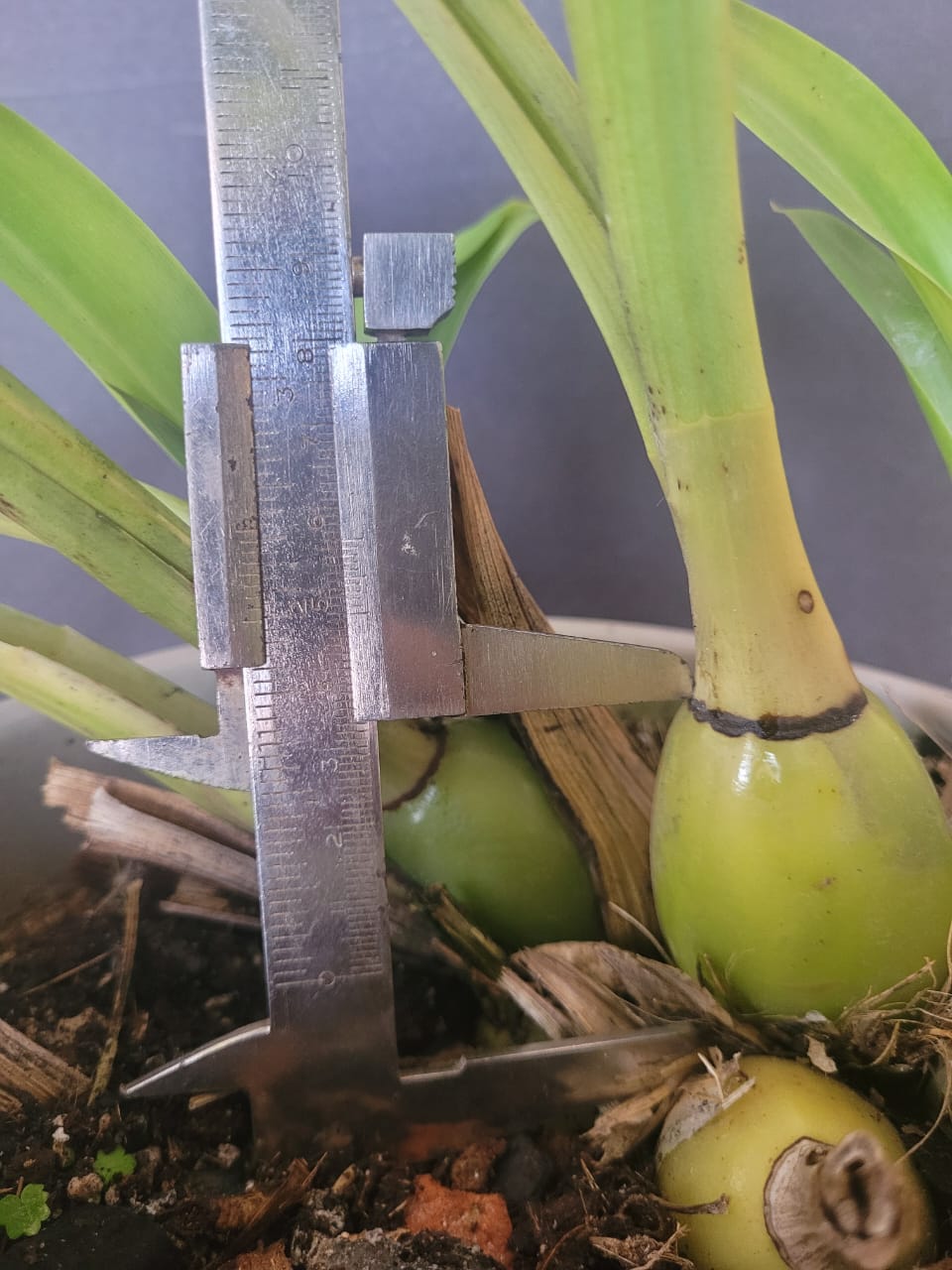 | 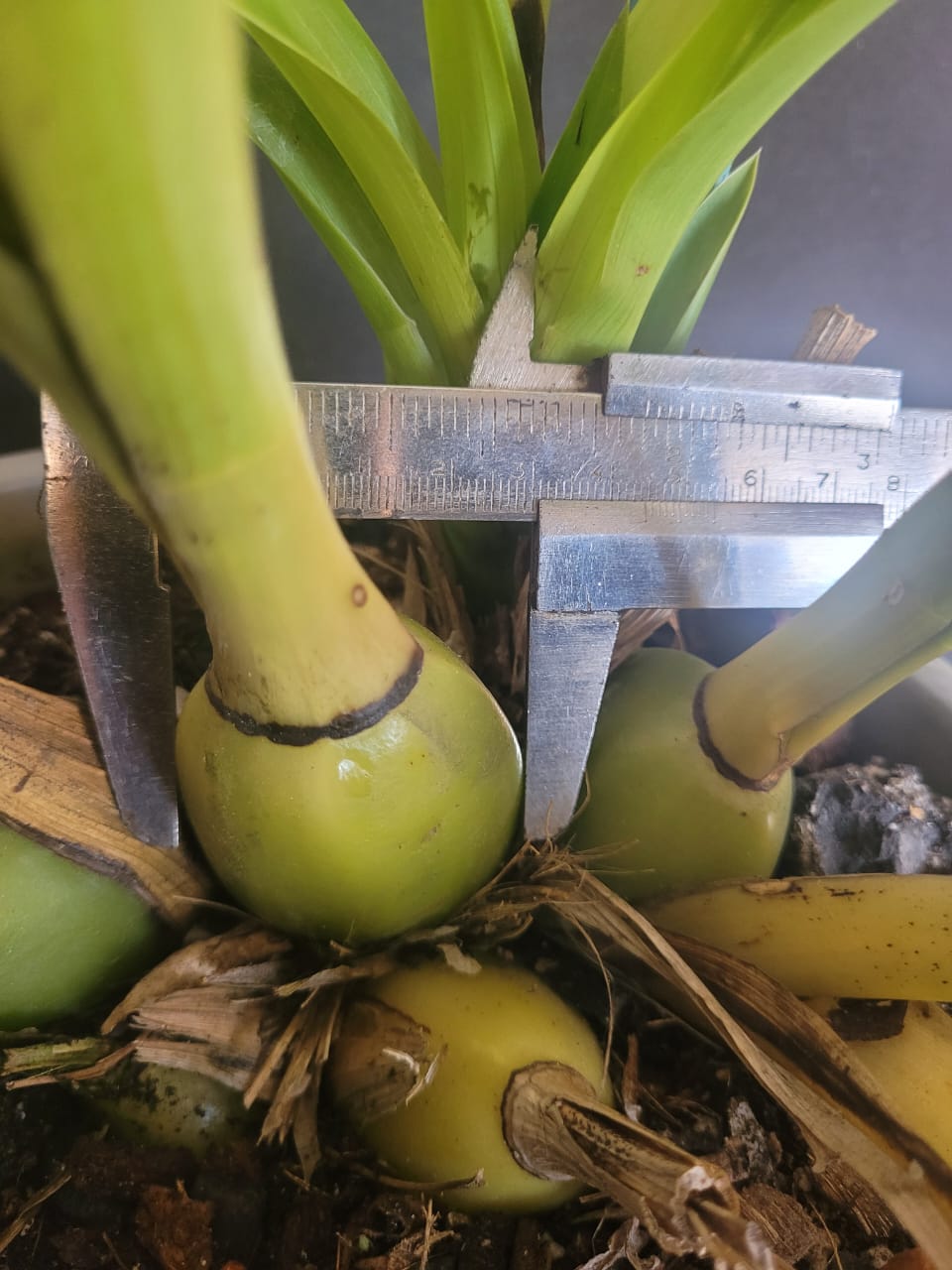 |
| --- | --- |
| A | B |
| 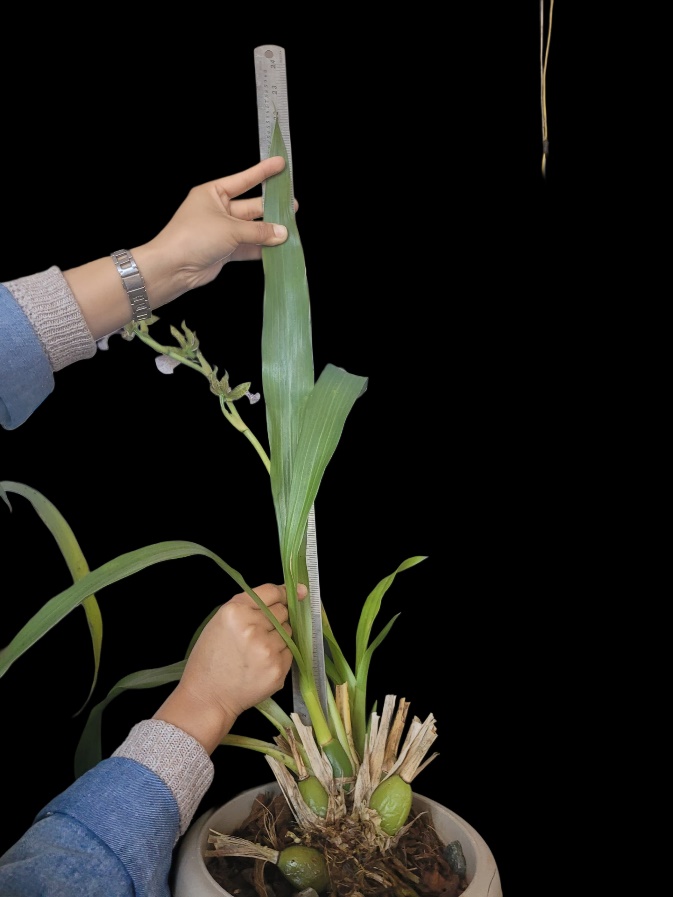 | 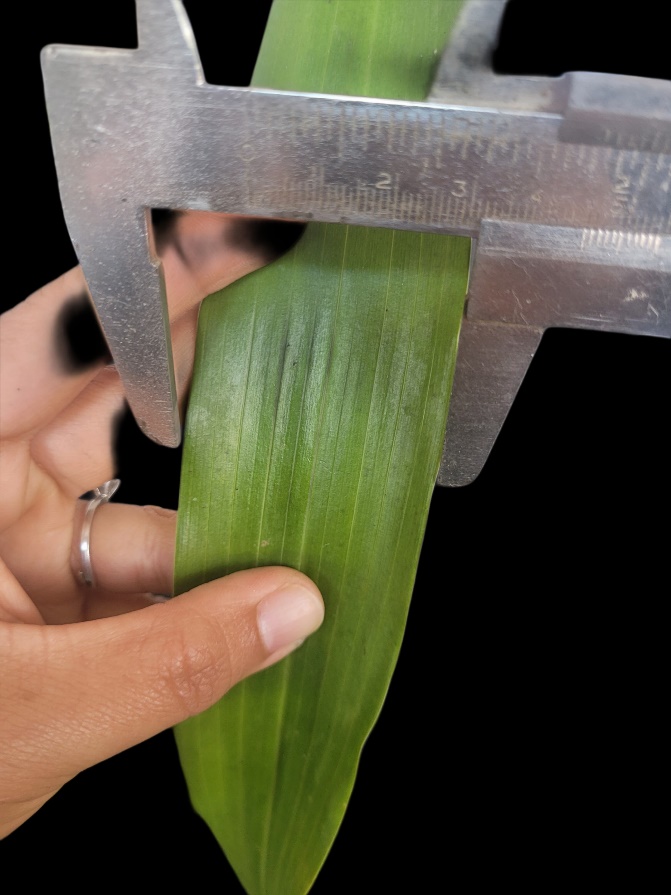 |
| C | D |
| 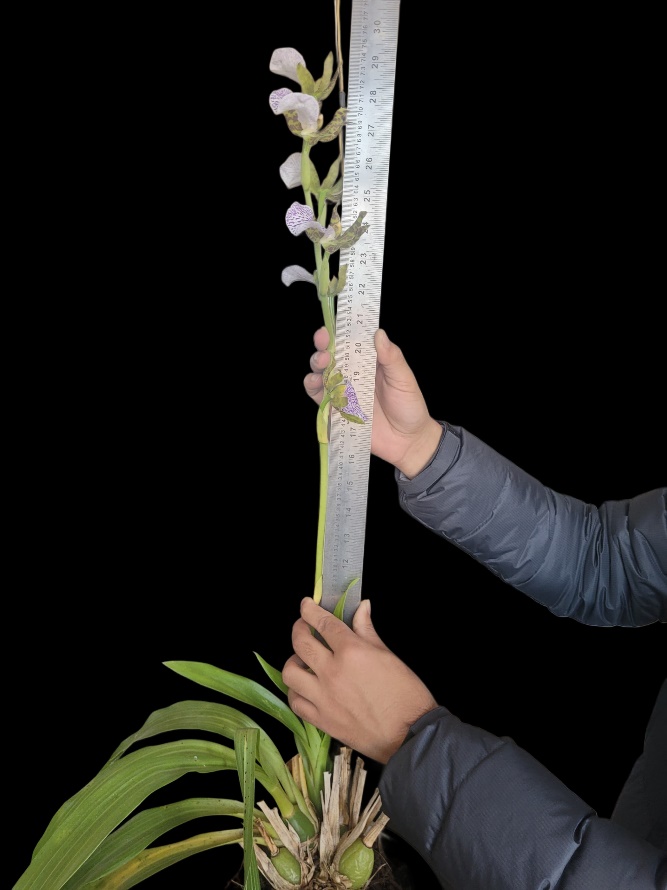 | 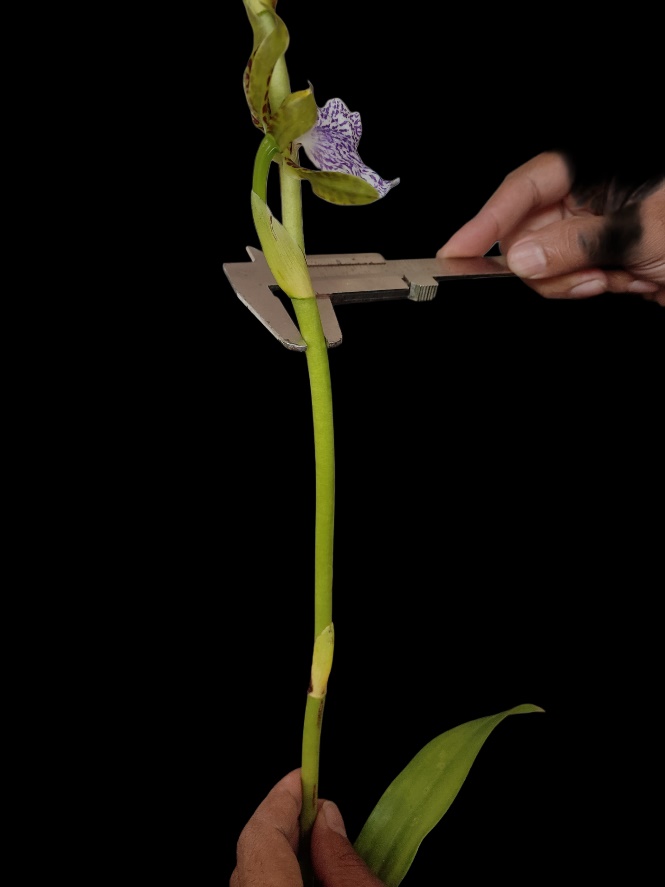 |
| E | F |
| 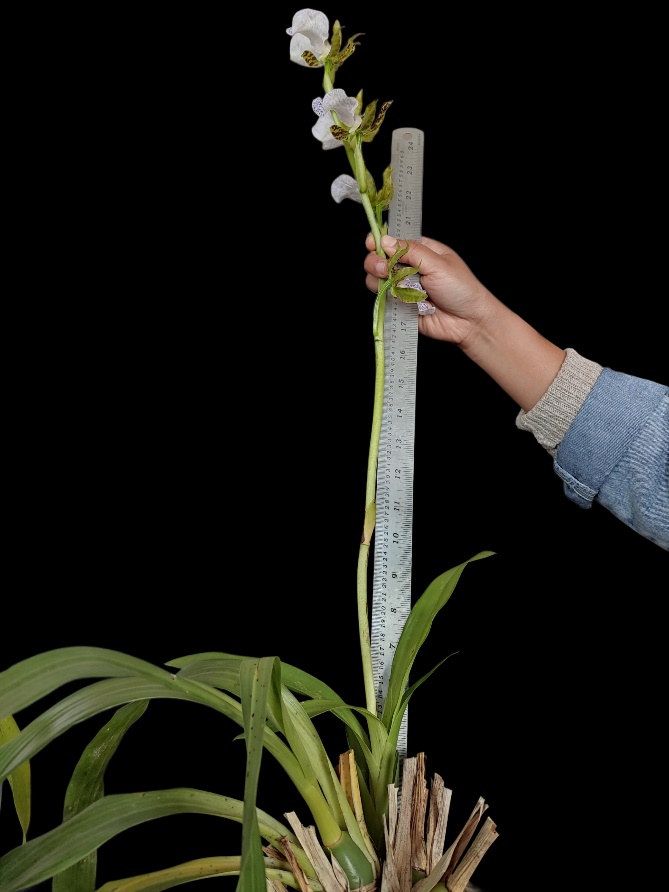 | 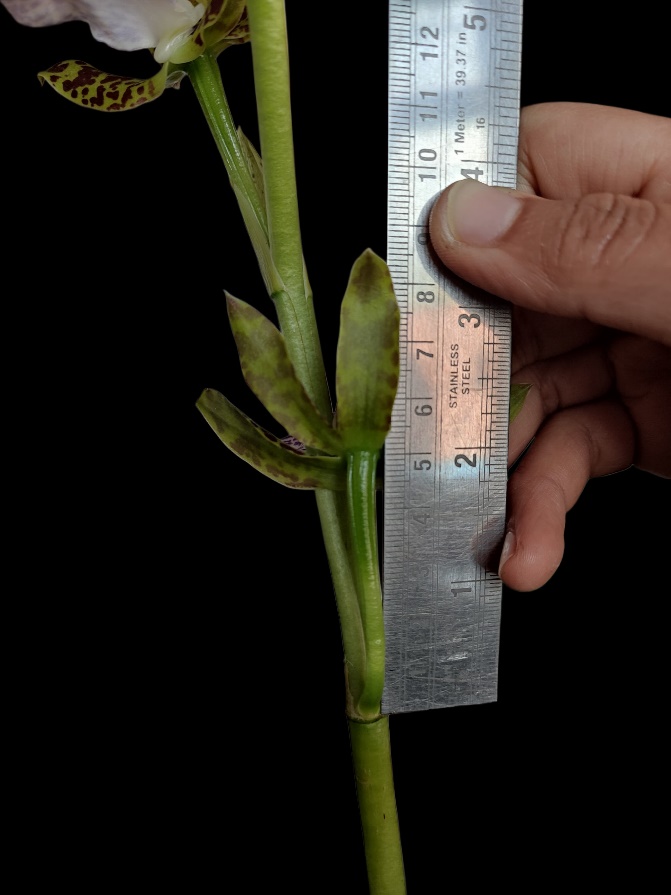 |
| G | H |
| 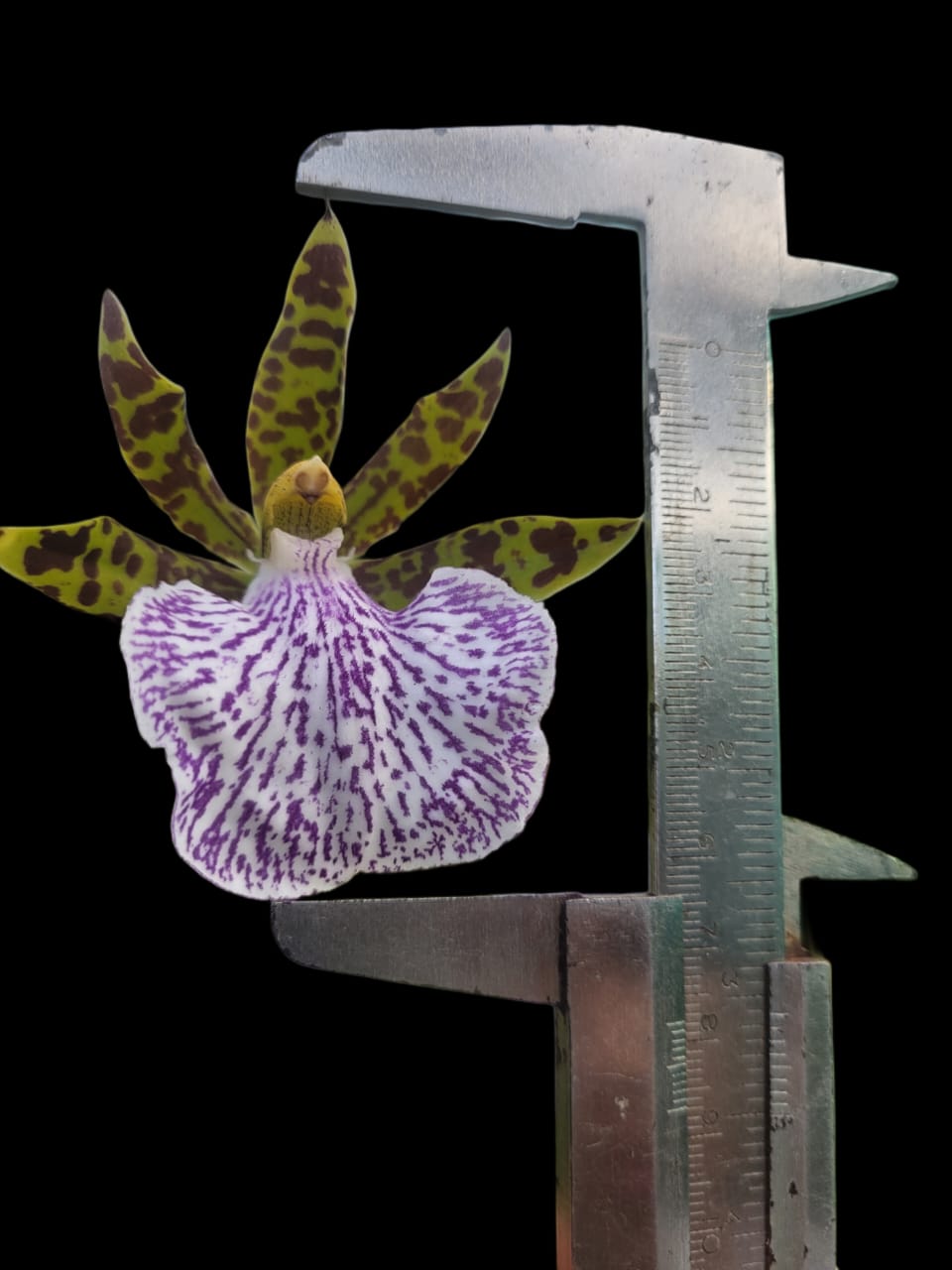 | 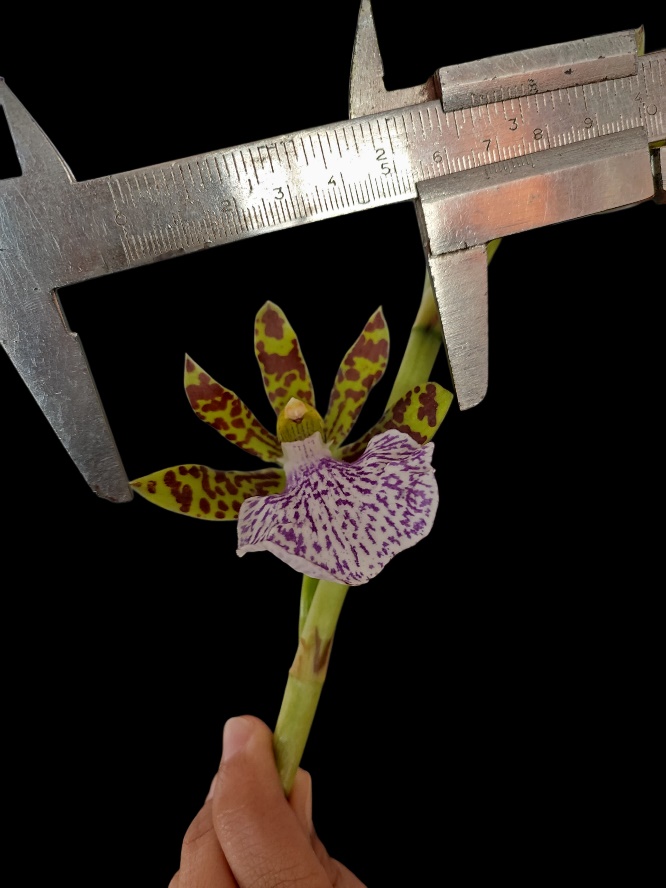 |
| I | J |

Supplementary figure 1: Method of recording (A) Bulb length, (B) bulb width, (C) leaf length, (D) leaf width, (E) spike length, (F) spike stem width, (G) peduncle length, (H) pedicel length, (I) floret length, and (J) floret width (for recording bulb, leaf and spike stem widths maximum width of respective parameter was recorded)
