# Supplementary material for: Potassium application enhances vegetative and reproductive yield of Zygopetalum maculatum and reduces post-flowering K depletion from storage organs of the orchid
Source: Sci Rep. 2025 Mar 29;15:10907. doi: 10.1038/s41598-025-89452-9 (PMC11954905; doi:10.1038/s41598-025-89452-9)
Supplement: Supplementary file 2 — Supplementary Information 2. [file 41598_2025_89452_MOESM2_ESM.docx]

**pH 0 D**

**Analysis of Variance Table**

| **Source of Variation<="" th="">** | **DF** | **Sum of Squares<="" th="">** | **Mean Squares<="" th="">** | **F-Calculated<="" th="">** | **Signficance<="" th="">** |
| --- | --- | --- | --- | --- | --- |
| Treatment | 5 | 0.007 | 0.001 | 0.010 | 0.99996 |
| Error | 18 | 2.348 | 0.130 |  |  |
| Total | 23 | 2.354 |  |  |  |

**pH 30 D**

**Analysis of Variance Table**

| **Source of Variation<="" th="">** | **DF** | **Sum of Squares<="" th="">** | **Mean Squares<="" th="">** | **F-Calculated<="" th="">** | **Signficance<="" th="">** |
| --- | --- | --- | --- | --- | --- |
| Treatment | 5 | 0.018 | 0.004 | 0.026 | 0.99961 |
| Error | 18 | 2.447 | 0.136 |  |  |
| Total | 23 | 2.465 |  |  |  |

**pH 90 D**

**Analysis of Variance Table**

| **Source of Variation<="" th="">** | **DF** | **Sum of Squares<="" th="">** | **Mean Squares<="" th="">** | **F-Calculated<="" th="">** | **Signficance<="" th="">** |
| --- | --- | --- | --- | --- | --- |
| Treatment | 5 | 0.192 | 0.038 | 2.782 | 0.04946 |
| Error | 18 | 0.249 | 0.014 |  |  |
| Total | 23 | 0.441 |  |  |  |

**pH 150 D**

**Analysis of Variance Table**

| **Source of Variation<="" th="">** | **DF** | **Sum of Squares<="" th="">** | **Mean Squares<="" th="">** | **F-Calculated<="" th="">** | **Signficance<="" th="">** |
| --- | --- | --- | --- | --- | --- |
| Treatment | 5 | 0.193 | 0.039 | 3.055 | 0.03608 |
| Error | 18 | 0.228 | 0.013 |  |  |
| Total | 23 | 0.421 |  |  |  |

**pH 210 D**

**Analysis of Variance Table**

| **Source of Variation<="" th="">** | **DF** | **Sum of Squares<="" th="">** | **Mean Squares<="" th="">** | **F-Calculated<="" th="">** | **Signficance<="" th="">** |
| --- | --- | --- | --- | --- | --- |
| Treatment | 5 | 0.219 | 0.044 | 2.805 | 0.04817 |
| Error | 18 | 0.281 | 0.016 |  |  |
| Total | 23 | 0.499 |  |  |  |

**pH 290 D**

**Analysis of Variance Table**

| **Source of Variation<="" th="">** | **DF** | **Sum of Squares<="" th="">** | **Mean Squares<="" th="">** | **F-Calculated<="" th="">** | **Signficance<="" th="">** |
| --- | --- | --- | --- | --- | --- |
| Treatment | 5 | 0.361 | 0.072 | 3.638 | 0.01891 |
| Error | 18 | 0.357 | 0.020 |  |  |
| Total | 23 | 0.718 |  |  |  |

**pH 370 D**

**Analysis of Variance Table**

| **Source of Variation<="" th="">** | **DF** | **Sum of Squares<="" th="">** | **Mean Squares<="" th="">** | **F-Calculated<="" th="">** | **Signficance<="" th="">** |
| --- | --- | --- | --- | --- | --- |
| Treatment | 5 | 0.535 | 0.107 | 4.689 | 0.00646 |
| Error | 18 | 0.411 | 0.023 |  |  |
| Total | 23 | 0.946 |  |  |  |

**pH 450 D**

**Analysis of Variance Table**

| **Source of Variation<="" th="">** | **DF** | **Sum of Squares<="" th="">** | **Mean Squares<="" th="">** | **F-Calculated<="" th="">** | **Signficance<="" th="">** |
| --- | --- | --- | --- | --- | --- |
| Treatment | 5 | 0.776 | 0.155 | 5.285 | 0.00368 |
| Error | 18 | 0.528 | 0.029 |  |  |
| Total | 23 | 1.304 |  |  |  |

**pH 650 D**

**Analysis of Variance Table**

| **Source of Variation<="" th="">** | **DF** | **Sum of Squares<="" th="">** | **Mean Squares<="" th="">** | **F-Calculated<="" th="">** | **Signficance<="" th="">** |
| --- | --- | --- | --- | --- | --- |
| Treatment | 5 | 1.113 | 0.223 | 12.960 | 0.00002 |
| Error | 18 | 0.309 | 0.017 |  |  |
| Total | 23 | 1.422 |  |  |  |

**Back Bulb (BB) Length**

**Analysis of Variance Table**

| **Source of Variation<="" th="">** | **DF** | **Sum of Squares<="" th="">** | **Mean Squares<="" th="">** | **F-Calculated<="" th="">** | **Signficance<="" th="">** |
| --- | --- | --- | --- | --- | --- |
| Treatment | 5 | 5.520 | 1.104 | 6.718 | 0.00107 |
| Error | 18 | 2.958 | 0.164 |  |  |
| Total | 23 | 8.478 |  |  |  |

**BB width**

**Analysis of Variance Table**

| **Source of Variation<="" th="">** | **DF** | **Sum of Squares<="" th="">** | **Mean Squares<="" th="">** | **F-Calculated<="" th="">** | **Signficance<="" th="">** |
| --- | --- | --- | --- | --- | --- |
| Treatment | 5 | 4.850 | 0.970 | 5.314 | 0.00358 |
| Error | 18 | 3.285 | 0.183 |  |  |
| Total | 23 | 8.135 |  |  |  |

**Leaf numbers**

**Analysis of Variance Table**

| **Source of Variation<="" th="">** | **DF** | **Sum of Squares<="" th="">** | **Mean Squares<="" th="">** | **F-Calculated<="" th="">** | **Signficance<="" th="">** |
| --- | --- | --- | --- | --- | --- |
| Treatment | 5 | 17.708 | 3.542 | 15.000 | 0.00001 |
| Error | 18 | 4.250 | 0.236 |  |  |
| Total | 23 | 21.958 |  |  |  |

**Leaf length**

**Analysis of Variance Table**

| **Source of Variation<="" th="">** | **DF** | **Sum of Squares<="" th="">** | **Mean Squares<="" th="">** | **F-Calculated<="" th="">** | **Signficance<="" th="">** |
| --- | --- | --- | --- | --- | --- |
| Treatment | 5 | 833.286 | 166.657 | 25.150 | 0.00000 |
| Error | 18 | 119.277 | 6.626 |  |  |
| Total | 23 | 952.563 |  |  |  |

**Leaf width**

**Analysis of Variance Table**

| **Source of Variation<="" th="">** | **DF** | **Sum of Squares<="" th="">** | **Mean Squares<="" th="">** | **F-Calculated<="" th="">** | **Signficance<="" th="">** |
| --- | --- | --- | --- | --- | --- |
| Treatment | 5 | 3.514 | 0.703 | 8.997 | 0.00020 |
| Error | 18 | 1.406 | 0.078 |  |  |
| Total | 23 | 4.921 |  |  |  |

**Spike number**

**Analysis of Variance Table**

| **Source of Variation<="" th="">** | **DF** | **Sum of Squares<="" th="">** | **Mean Squares<="" th="">** | **F-Calculated<="" th="">** | **Signficance<="" th="">** |
| --- | --- | --- | --- | --- | --- |
| Treatment | 5 | 4.375 | 0.875 | 4.846 | 0.00555 |
| Error | 18 | 3.250 | 0.181 |  |  |
| Total | 23 | 7.625 |  |  |  |

**Spike length**

**Analysis of Variance Table**

| **Source of Variation<="" th="">** | **DF** | **Sum of Squares<="" th="">** | **Mean Squares<="" th="">** | **F-Calculated<="" th="">** | **Signficance<="" th="">** |
| --- | --- | --- | --- | --- | --- |
| Treatment | 5 | 871.792 | 174.358 | 13.256 | 0.00002 |
| Error | 18 | 236.752 | 13.153 |  |  |
| Total | 23 | 1,108.544 |  |  |  |

**Spike stem width**

**Analysis of Variance Table**

| **Source of Variation<="" th="">** | **DF** | **Sum of Squares<="" th="">** | **Mean Squares<="" th="">** | **F-Calculated<="" th="">** | **Signficance<="" th="">** |
| --- | --- | --- | --- | --- | --- |
| Treatment | 5 | 0.273 | 0.055 | 30.029 | 0.00000 |
| Error | 18 | 0.033 | 0.002 |  |  |
| Total | 23 | 0.306 |  |  |  |

**Peduncle length**

**Analysis of Variance Table**

| **Source of Variation<="" th="">** | **DF** | **Sum of Squares<="" th="">** | **Mean Squares<="" th="">** | **F-Calculated<="" th="">** | **Signficance<="" th="">** |
| --- | --- | --- | --- | --- | --- |
| Treatment | 5 | 188.116 | 37.623 | 9.342 | 0.00016 |
| Error | 18 | 72.488 | 4.027 |  |  |
| Total | 23 | 260.604 |  |  |  |

**Pedicel length**

**Analysis of Variance Table**

| **Source of Variation<="" th="">** | **DF** | **Sum of Squares<="" th="">** | **Mean Squares<="" th="">** | **F-Calculated<="" th="">** | **Signficance<="" th="">** |
| --- | --- | --- | --- | --- | --- |
| Treatment | 5 | 8.776 | 1.755 | 18.424 | 0.00000 |
| Error | 18 | 1.715 | 0.095 |  |  |
| Total | 23 | 10.490 |  |  |  |

**Florets per spike**

**Analysis of Variance Table**

| **Source of Variation<="" th="">** | **DF** | **Sum of Squares<="" th="">** | **Mean Squares<="" th="">** | **F-Calculated<="" th="">** | **Signficance<="" th="">** |
| --- | --- | --- | --- | --- | --- |
| Treatment | 5 | 9.333 | 1.867 | 7.467 | 0.00060 |
| Error | 18 | 4.500 | 0.250 |  |  |
| Total | 23 | 13.833 |  |  |  |

**Floret length**

**Analysis of Variance Table**

| **Source of Variation<="" th="">** | **DF** | **Sum of Squares<="" th="">** | **Mean Squares<="" th="">** | **F-Calculated<="" th="">** | **Signficance<="" th="">** |
| --- | --- | --- | --- | --- | --- |
| Treatment | 5 | 9.215 | 1.843 | 10.517 | 0.00008 |
| Error | 18 | 3.154 | 0.175 |  |  |
| Total | 23 | 12.370 |  |  |  |

**Floret width**

**Analysis of Variance Table**

| **Source of Variation<="" th="">** | **DF** | **Sum of Squares<="" th="">** | **Mean Squares<="" th="">** | **F-Calculated<="" th="">** | **Signficance<="" th="">** |
| --- | --- | --- | --- | --- | --- |
| Treatment | 5 | 5.423 | 1.085 | 6.653 | 0.00113 |
| Error | 18 | 2.935 | 0.163 |  |  |
| Total | 23 | 8.358 |  |  |  |

**Leaf moisture**

**Analysis of Variance Table**

| **Source of Variation<="" th="">** | **DF** | **Sum of Squares<="" th="">** | **Mean Squares<="" th="">** | **F-Calculated<="" th="">** | **Signficance<="" th="">** |
| --- | --- | --- | --- | --- | --- |
| Treatment | 5 | 322.418 | 64.484 | 3.281 | 0.02795 |
| Error | 18 | 353.729 | 19.652 |  |  |
| Total | 23 | 676.147 |  |  |  |

**BB moisture**

**Analysis of Variance Table**

| **Source of Variation<="" th="">** | **DF** | **Sum of Squares<="" th="">** | **Mean Squares<="" th="">** | **F-Calculated<="" th="">** | **Signficance<="" th="">** |
| --- | --- | --- | --- | --- | --- |
| Treatment | 5 | 84.892 | 16.978 | 2.961 | 0.04021 |
| Error | 18 | 103.226 | 5.735 |  |  |
| Total | 23 | 188.118 |  |  |  |

**NB moisture**

**Analysis of Variance Table**

| **Source of Variation<="" th="">** | **DF** | **Sum of Squares<="" th="">** | **Mean Squares<="" th="">** | **F-Calculated<="" th="">** | **Signficance<="" th="">** |
| --- | --- | --- | --- | --- | --- |
| Treatment | 5 | 21.450 | 4.290 | 1.228 | 0.33666 |
| Error | 18 | 62.890 | 3.494 |  |  |
| Total | 23 | 84.340 |  |  |  |

**Flower moisture**

**Analysis of Variance Table**

| **Source of Variation<="" th="">** | **DF** | **Sum of Squares<="" th="">** | **Mean Squares<="" th="">** | **F-Calculated<="" th="">** | **Signficance<="" th="">** |
| --- | --- | --- | --- | --- | --- |
| Treatment | 5 | 4.835 | 0.967 | 0.256 | 0.93134 |
| Error | 18 | 68.062 | 3.781 |  |  |
| Total | 23 | 72.896 |  |  |  |

**Root moisture**

**Analysis of Variance Table**

| **Source of Variation<="" th="">** | **DF** | **Sum of Squares<="" th="">** | **Mean Squares<="" th="">** | **F-Calculated<="" th="">** | **Signficance<="" th="">** |
| --- | --- | --- | --- | --- | --- |
| Treatment | 5 | 208.928 | 41.786 | 2.822 | 0.04720 |
| Error | 18 | 266.507 | 14.806 |  |  |
| Total | 23 | 475.435 |  |  |  |

**Leaf K before flowering**

**Analysis of Variance Table**

| **Source of Variation<="" th="">** | **DF** | **Sum of Squares<="" th="">** | **Mean Squares<="" th="">** | **F-Calculated<="" th="">** | **Signficance<="" th="">** |
| --- | --- | --- | --- | --- | --- |
| Treatment | 5 | 0.817 | 0.163 | 24.264 | 0.00000 |
| Error | 18 | 0.121 | 0.007 |  |  |
| Total | 23 | 0.938 |  |  |  |

**BB K before flowering**

**Analysis of Variance Table**

| **Source of Variation<="" th="">** | **DF** | **Sum of Squares<="" th="">** | **Mean Squares<="" th="">** | **F-Calculated<="" th="">** | **Signficance<="" th="">** |
| --- | --- | --- | --- | --- | --- |
| Treatment | 5 | 0.890 | 0.178 | 43.302 | 0.00000 |
| Error | 18 | 0.074 | 0.004 |  |  |
| Total | 23 | 0.964 |  |  |  |

**NB K before flowering**

**Analysis of Variance Table**

| **Source of Variation<="" th="">** | **DF** | **Sum of Squares<="" th="">** | **Mean Squares<="" th="">** | **F-Calculated<="" th="">** | **Signficance<="" th="">** |
| --- | --- | --- | --- | --- | --- |
| Treatment | 5 | 0.177 | 0.035 | 31.066 | 0.00000 |
| Error | 18 | 0.021 | 0.001 |  |  |
| Total | 23 | 0.198 |  |  |  |

**Root K before flowering**

**Analysis of Variance Table**

| **Source of Variation<="" th="">** | **DF** | **Sum of Squares<="" th="">** | **Mean Squares<="" th="">** | **F-Calculated<="" th="">** | **Signficance<="" th="">** |
| --- | --- | --- | --- | --- | --- |
| Treatment | 5 | 0.854 | 0.171 | 18.621 | 0.00000 |
| Error | 18 | 0.165 | 0.009 |  |  |
| Total | 23 | 1.020 |  |  |  |

**Leaf K after flowering**

**Analysis of Variance Table**

| **Source of Variation<="" th="">** | **DF** | **Sum of Squares<="" th="">** | **Mean Squares<="" th="">** | **F-Calculated<="" th="">** | **Signficance<="" th="">** |
| --- | --- | --- | --- | --- | --- |
| Treatment | 5 | 1.253 | 0.251 | 46.314 | 0.00000 |
| Error | 18 | 0.097 | 0.005 |  |  |
| Total | 23 | 1.351 |  |  |  |

**BB K after flowering**

**Analysis of Variance Table**

| **Source of Variation<="" th="">** | **DF** | **Sum of Squares<="" th="">** | **Mean Squares<="" th="">** | **F-Calculated<="" th="">** | **Signficance<="" th="">** |
| --- | --- | --- | --- | --- | --- |
| Treatment | 5 | 1.335 | 0.267 | 94.352 | 0.00000 |
| Error | 18 | 0.051 | 0.003 |  |  |
| Total | 23 | 1.386 |  |  |  |

**NB K after flowering**

**Analysis of Variance Table**

| **Source of Variation<="" th="">** | **DF** | **Sum of Squares<="" th="">** | **Mean Squares<="" th="">** | **F-Calculated<="" th="">** | **Signficance<="" th="">** |
| --- | --- | --- | --- | --- | --- |
| Treatment | 5 | 0.253 | 0.051 | 35.542 | 0.00000 |
| Error | 18 | 0.026 | 0.001 |  |  |
| Total | 23 | 0.279 |  |  |  |

**Root K after flowering**

**Analysis of Variance Table**

| **Source of Variation<="" th="">** | **DF** | **Sum of Squares<="" th="">** | **Mean Squares<="" th="">** | **F-Calculated<="" th="">** | **Signficance<="" th="">** |
| --- | --- | --- | --- | --- | --- |
| Treatment | 5 | 1.343 | 0.269 | 48.498 | 0.00000 |
| Error | 18 | 0.100 | 0.006 |  |  |
| Total | 23 | 1.442 |  |  |  |

**Leaf K reduction**

**Analysis of Variance Table**

| **Source of Variation<="" th="">** | **DF** | **Sum of Squares<="" th="">** | **Mean Squares<="" th="">** | **F-Calculated<="" th="">** | **Signficance<="" th="">** |
| --- | --- | --- | --- | --- | --- |
| Treatment | 5 | 728.819 | 145.764 | 40.379 | 0.00000 |
| Error | 18 | 64.978 | 3.610 |  |  |
| Total | 23 | 793.798 |  |  |  |

**BB K reduction**

**Analysis of Variance Table**

| **Source of Variation<="" th="">** | **DF** | **Sum of Squares<="" th="">** | **Mean Squares<="" th="">** | **F-Calculated<="" th="">** | **Signficance<="" th="">** |
| --- | --- | --- | --- | --- | --- |
| Treatment | 5 | 4,294.925 | 858.985 | 136.383 | 0.00000 |
| Error | 18 | 113.370 | 6.298 |  |  |
| Total | 23 | 4,408.295 |  |  |  |

**Root K reduction**

**Analysis of Variance Table**

| **Source of Variation<="" th="">** | **DF** | **Sum of Squares<="" th="">** | **Mean Squares<="" th="">** | **F-Calculated<="" th="">** | **Signficance<="" th="">** |
| --- | --- | --- | --- | --- | --- |
| Treatment | 5 | 1,751.093 | 350.219 | 84.439 | 0.00000 |
| Error | 18 | 74.656 | 4.148 |  |  |
| Total | 23 | 1,825.749 |  |  |  |

**Water extractable K (WEK)**

**Analysis of Variance Table**

| **Source of Variation<="" th="">** | **DF** | **Sum of Squares<="" th="">** | **Mean Squares<="" th="">** | **F-Calculated<="" th="">** | **Signficance<="" th="">** |
| --- | --- | --- | --- | --- | --- |
| Treatment | 5 | 27,863.286 | 5,572.657 | 186.036 | 0.00000 |
| Error | 18 | 539.184 | 29.955 |  |  |
| Total | 23 | 28,402.470 |  |  |  |

**Dehydrogenase activity (DHA)**

**Analysis of Variance Table**

| **Source of Variation<="" th="">** | **DF** | **Sum of Squares<="" th="">** | **Mean Squares<="" th="">** | **F-Calculated<="" th="">** | **Signficance<="" th="">** |
| --- | --- | --- | --- | --- | --- |
| Treatment | 5 | 3.598 | 0.720 | 31.461 | 0.00000 |
| Error | 18 | 0.412 | 0.023 |  |  |
| Total | 23 | 4.009 |  |  |  |

**Fresh flower biomass**

**Analysis of Variance Table**

| **Source of Variation<="" th="">** | **DF** | **Sum of Squares<="" th="">** | **Mean Squares<="" th="">** | **F-Calculated<="" th="">** | **Signficance<="" th="">** |
| --- | --- | --- | --- | --- | --- |
| Treatment | 5 | 648.334 | 129.667 | 32.272 | 0.00000 |
| Error | 18 | 72.323 | 4.018 |  |  |
| Total | 23 | 720.657 |  |  |  |

**Dry flower biomass**

**Analysis of Variance Table**

| **Source of Variation<="" th="">** | **DF** | **Sum of Squares<="" th="">** | **Mean Squares<="" th="">** | **F-Calculated<="" th="">** | **Signficance<="" th="">** |
| --- | --- | --- | --- | --- | --- |
| Treatment | 5 | 3.103 | 0.621 | 56.475 | 0.00000 |
| Error | 18 | 0.198 | 0.011 |  |  |
| Total | 23 | 3.300 |  |  |  |

**New shoot numbers**

**Analysis of Variance Table**

| **Source of Variation<="" th="">** | **DF** | **Sum of Squares<="" th="">** | **Mean Squares<="" th="">** | **F-Calculated<="" th="">** | **Signficance<="" th="">** |
| --- | --- | --- | --- | --- | --- |
| Treatment | 5 | 3.833 | 0.767 | 3.943 | 0.01369 |
| Error | 18 | 3.500 | 0.194 |  |  |
| Total | 23 | 7.333 |  |  |  |

**K in flowers**

**Analysis of Variance Table**

| **Source of Variation<="" th="">** | **DF** | **Sum of Squares<="" th="">** | **Mean Squares<="" th="">** | **F-Calculated<="" th="">** | **Signficance<="" th="">** |
| --- | --- | --- | --- | --- | --- |
| Treatment | 5 | 0.351 | 0.070 | 19.672 | 0.00000 |
| Error | 18 | 0.064 | 0.004 |  |  |
| Total | 23 | 0.415 |  |  |  |

**K uptake in flower**

**Analysis of Variance Table**

| **Source of Variation<="" th="">** | **DF** | **Sum of Squares<="" th="">** | **Mean Squares<="" th="">** | **F-Calculated<="" th="">** | **Signficance<="" th="">** |
| --- | --- | --- | --- | --- | --- |
| Treatment | 5 | 7,871.111 | 1,574.222 | 19.499 | 0.00000 |
| Error | 18 | 1,453.217 | 80.734 |  |  |
| Total | 23 | 9,324.328 |  |  |  |

**Vase life**

**Analysis of Variance Table**

| **Source of Variation<="" th="">** | **DF** | **Sum of Squares<="" th="">** | **Mean Squares<="" th="">** | **F-Calculated<="" th="">** | **Signficance<="" th="">** |
| --- | --- | --- | --- | --- | --- |
| Treatment | 5 | 8.959 | 1.792 | 8.462 | 0.00029 |
| Error | 18 | 3.811 | 0.212 |  |  |
| Total | 23 | 12.771 |  |  |  |

**TABLES OF MEAN, STANDARD ERRORS AND LSD**

| **Treatment** | **pH 0 D** | | **pH 30 D** | | **pH 90 D** | | **pH 150 D** | | **pH 210 D** | | **pH 290 D** | | **pH 370 D** | | **pH 450 D** | |
| --- | --- | --- | --- | --- | --- | --- | --- | --- | --- | --- | --- | --- | --- | --- | --- | --- |
|  | **Mean** | **S.E.** | **Mean** | **S.E.** | **Mean** | **S.E.** | **Mean** | **S.E.** | **Mean** | **S.E.** | **Mean** | **S.E.** | **Mean** | **S.E.** | **Mean** | **S.E.** |
| **1** | 6.933 | 0.203 | 7.063 | 0.205 | 6.830 | 0.049 | 6.741 | 0.038 | 6.690 | 0.057 | 6.540 | 0.093 | 6.436 | 0.111 | 6.280 | 0.112 |
| **2** | 6.918 | 0.135 | 7.038 | 0.138 | 6.820 | 0.044 | 6.768 | 0.048 | 6.708 | 0.058 | 6.590 | 0.072 | 6.470 | 0.071 | 6.311 | 0.110 |
| **3** | 6.938 | 0.175 | 7.053 | 0.180 | 6.812 | 0.049 | 6.791 | 0.058 | 6.750 | 0.047 | 6.630 | 0.056 | 6.520 | 0.055 | 6.400 | 0.068 |
| **4** | 6.911 | 0.192 | 7.031 | 0.195 | 6.843 | 0.064 | 6.780 | 0.080 | 6.760 | 0.069 | 6.640 | 0.064 | 6.540 | 0.070 | 6.430 | 0.062 |
| **5** | 6.948 | 0.191 | 7.089 | 0.198 | 6.872 | 0.073 | 6.810 | 0.044 | 6.780 | 0.077 | 6.710 | 0.073 | 6.659 | 0.073 | 6.569 | 0.086 |
| **6** | 6.960 | 0.180 | 7.109 | 0.183 | 7.070 | 0.067 | 7.012 | 0.058 | 6.980 | 0.063 | 6.921 | 0.059 | 6.879 | 0.059 | 6.811 | 0.058 |
| LSD | N/A |  | N/A |  | 0.176 |  | 0.168 |  | 0.187 |  | 0.211 |  | 0.226 |  | 0.257 |  |
| SE(m) | 0.181 |  | 0.184 |  | 0.059 |  | 0.056 |  | 0.062 |  | 0.070 |  | 0.076 |  | 0.086 |  |
| SE(d) | 0.255 |  | 0.261 |  | 0.083 |  | 0.080 |  | 0.088 |  | 0.100 |  | 0.107 |  | 0.121 |  |
| C.V. | 5.208 |  | 5.219 |  | 1.711 |  | 1.650 |  | 1.842 |  | 2.111 |  | 2.295 |  | 2.650 |  |

| **Treatment** | **pH 650 D** | | **BB length** | | **BB Width** | | **Leaf numbers** | | **Leaf length** | | **Leaf width** | | **Spike number** | | **Spike length** | |
| --- | --- | --- | --- | --- | --- | --- | --- | --- | --- | --- | --- | --- | --- | --- | --- | --- |
|  | **Mean** | **S.E.** | **Mean** | **S.E.** | **Mean** | **S.E.** | **Mean** | **S.E.** | **Mean** | **S.E.** | **Mean** | **S.E.** | **Mean** | **S.E.** | **Mean** | **S.E.** |
| **1** | 6.070 | 0.083 | 5.191 | 0.135 | 4.689 | 0.224 | 6.500 | 0.289 | 38.009 | 1.723 | 3.913 | 0.175 | 0.750 | 0.250 | 53.113 | 1.924 |
| **2** | 6.118 | 0.068 | 5.511 | 0.196 | 4.672 | 0.167 | 6.750 | 0.250 | 37.923 | 0.414 | 3.988 | 0.083 | 1.000 | 0.000 | 55.608 | 2.177 |
| **3** | 6.206 | 0.051 | 5.591 | 0.281 | 5.101 | 0.160 | 7.250 | 0.250 | 42.275 | 1.528 | 4.089 | 0.151 | 1.250 | 0.250 | 60.193 | 0.870 |
| **4** | 6.291 | 0.061 | 5.810 | 0.176 | 5.171 | 0.211 | 7.500 | 0.289 | 47.310 | 0.451 | 4.251 | 0.159 | 1.500 | 0.289 | 64.410 | 2.202 |
| **5** | 6.380 | 0.069 | 6.105 | 0.251 | 5.679 | 0.260 | 8.250 | 0.250 | 50.725 | 1.318 | 4.559 | 0.088 | 1.750 | 0.250 | 65.318 | 1.096 |
| **6** | 6.721 | 0.057 | 6.689 | 0.129 | 5.858 | 0.241 | 9.000 | 0.000 | 52.995 | 1.588 | 5.020 | 0.154 | 2.000 | 0.000 | 70.890 | 2.117 |
| LSD | 0.196 |  | 0.607 |  | 0.640 |  | 0.727 |  | 3.854 |  | 0.418 |  | 0.636 |  | 5.429 |  |
| SE(m) | 0.066 |  | 0.203 |  | 0.214 |  | 0.243 |  | 1.287 |  | 0.140 |  | 0.212 |  | 1.813 |  |
| SE(d) | 0.093 |  | 0.287 |  | 0.302 |  | 0.344 |  | 1.820 |  | 0.198 |  | 0.300 |  | 2.564 |  |
| C.V. | 2.081 |  | 6.970 |  | 8.224 |  | 6.443 |  | 5.737 |  | 6.495 |  | 30.903 |  | 5.889 |  |

| **Treatment** | **Spike stem width** | | **Peduncle length** | | **Pedicel length** | | Florets per spike | | **Floret length** | | **Floret width** | | **Leaf moisture** | | **BB moisture** | |
| --- | --- | --- | --- | --- | --- | --- | --- | --- | --- | --- | --- | --- | --- | --- | --- | --- |
|  | **Mean** | **S.E.** | **Mean** | **S.E.** | **Mean** | **S.E.** | **Mean** | **S.E.** | **Mean** | **S.E.** | **Mean** | **S.E.** | **Mean** | **S.E.** | **Mean** | **S.E.** |
| **1** | 0.622 | 0.025 | 28.183 | 1.172 | 4.518 | 0.180 | 4.500 | 0.289 | 6.313 | 0.248 | 5.518 | 0.236 | 77.340 | 2.286 | 85.200 | 1.155 |
| **2** | 0.772 | 0.028 | 31.388 | 1.135 | 4.973 | 0.129 | 5.000 | 0.000 | 6.505 | 0.191 | 6.113 | 0.183 | 79.590 | 2.418 | 87.700 | 2.104 |
| **3** | 0.799 | 0.010 | 31.508 | 1.047 | 5.555 | 0.158 | 5.250 | 0.250 | 6.972 | 0.213 | 6.159 | 0.204 | 82.103 | 2.224 | 88.400 | 0.755 |
| **4** | 0.851 | 0.032 | 34.315 | 1.107 | 5.788 | 0.136 | 5.750 | 0.250 | 7.240 | 0.178 | 6.460 | 0.180 | 82.210 | 1.961 | 87.600 | 0.843 |
| **5** | 0.886 | 0.010 | 35.785 | 0.782 | 6.013 | 0.184 | 5.500 | 0.289 | 7.313 | 0.235 | 6.540 | 0.227 | 85.710 | 2.484 | 90.300 | 0.981 |
| **6** | 0.965 | 0.008 | 36.098 | 0.668 | 6.278 | 0.129 | 6.500 | 0.289 | 8.218 | 0.179 | 7.079 | 0.173 | 88.415 | 1.855 | 90.900 | 0.774 |
| LSD | 0.064 |  | 3.004 |  | 0.462 |  | 0.749 |  | 0.627 |  | 0.604 |  | 6.637 |  | 3.585 |  |
| SE(m) | 0.021 |  | 1.003 |  | 0.154 |  | 0.250 |  | 0.209 |  | 0.202 |  | 2.217 |  | 1.197 |  |
| SE(d) | 0.030 |  | 1.419 |  | 0.218 |  | 0.354 |  | 0.296 |  | 0.286 |  | 3.135 |  | 1.693 |  |
| C.V. | 5.232 |  | 6.103 |  | 5.591 |  | 9.231 |  | 5.902 |  | 6.398 |  | 5.369 |  | 2.711 |  |

| **Treatment** | **NB moisture** | | **Flower moisture** | | **Root moisture** | | **Leaf K before flowering** | | **BB K before flowering** | | **NB K before flowering** | | **Root K before flowering** | | **Leaf K after flowering** | |
| --- | --- | --- | --- | --- | --- | --- | --- | --- | --- | --- | --- | --- | --- | --- | --- | --- |
|  | **Mean** | **S.E.** | **Mean** | **S.E.** | **Mean** | **S.E.** | **Mean** | **S.E.** | **Mean** | **S.E.** | **Mean** | **S.E.** | **Mean** | **S.E.** | **Mean** | **S.E.** |
| **1** | 88.400 | 1.199 | 91.808 | 1.251 | 73.513 | 2.610 | 1.160 | 0.031 | 0.932 | 0.024 | 0.512 | 0.013 | 1.359 | 0.036 | 0.982 | 0.025 |
| **2** | 88.498 | 0.964 | 92.293 | 1.008 | 75.188 | 2.385 | 1.310 | 0.032 | 0.949 | 0.024 | 0.561 | 0.014 | 1.611 | 0.041 | 1.120 | 0.028 |
| **3** | 88.690 | 0.751 | 92.513 | 0.798 | 76.113 | 2.190 | 1.460 | 0.041 | 1.140 | 0.031 | 0.584 | 0.016 | 1.719 | 0.052 | 1.309 | 0.037 |
| **4** | 90.402 | 0.868 | 92.898 | 0.895 | 79.685 | 1.156 | 1.560 | 0.042 | 1.150 | 0.030 | 0.651 | 0.017 | 1.819 | 0.049 | 1.473 | 0.040 |
| **5** | 90.200 | 0.980 | 92.108 | 1.006 | 78.583 | 1.238 | 1.580 | 0.045 | 1.290 | 0.035 | 0.660 | 0.017 | 1.881 | 0.049 | 1.496 | 0.038 |
| **6** | 90.599 | 0.772 | 93.110 | 0.799 | 82.310 | 1.429 | 1.720 | 0.052 | 1.490 | 0.044 | 0.779 | 0.023 | 1.910 | 0.057 | 1.647 | 0.048 |
| LSD | N/A |  | N/A |  | 5.761 |  | 0.123 |  | 0.096 |  | 0.051 |  | 0.143 |  | 0.110 |  |
| SE(m) | 0.935 |  | 0.972 |  | 1.924 |  | 0.041 |  | 0.032 |  | 0.017 |  | 0.048 |  | 0.037 |  |
| SE(d) | 1.322 |  | 1.375 |  | 2.721 |  | 0.058 |  | 0.045 |  | 0.024 |  | 0.068 |  | 0.052 |  |
| C.V. | 2.089 |  | 2.103 |  | 4.961 |  | 5.602 |  | 5.536 |  | 5.405 |  | 5.581 |  | 5.498 |  |

| **Treatment** | **BB K after flowering** | | **NB K after flowering** | | **Root K after flowering** | | **Leaf K reduction** | | **BB K reduction** | | **Root K reduction** | | **Water extractable K** | | **DHA** | |
| --- | --- | --- | --- | --- | --- | --- | --- | --- | --- | --- | --- | --- | --- | --- | --- | --- |
|  | **Mean** | **S.E.** | **Mean** | **S.E.** | **Mean** | **S.E.** | **Mean** | **S.E.** | **Mean** | **S.E.** | **Mean** | **S.E.** | **Mean** | **S.E.** | **Mean** | **S.E.** |
| **1** | 0.634 | 0.019 | 0.509 | 0.014 | 0.974 | 0.024 | 18.085 | 0.121 | 47.116 | 0.646 | 39.526 | 1.503 | 40.100 | 0.979 | 4.680 | 0.103 |
| **2** | 0.674 | 0.021 | 0.572 | 0.018 | 1.311 | 0.033 | 16.936 | 0.987 | 40.849 | 0.754 | 22.902 | 0.958 | 78.300 | 2.159 | 4.890 | 0.053 |
| **3** | 0.793 | 0.020 | 0.591 | 0.015 | 1.422 | 0.043 | 11.529 | 1.500 | 43.781 | 1.606 | 20.951 | 1.595 | 99.900 | 2.881 | 5.009 | 0.080 |
| **4** | 0.958 | 0.031 | 0.655 | 0.022 | 1.550 | 0.035 | 5.886 | 0.988 | 20.170 | 1.494 | 17.358 | 0.531 | 117.100 | 2.643 | 5.430 | 0.052 |
| **5** | 1.092 | 0.026 | 0.676 | 0.017 | 1.610 | 0.040 | 5.581 | 1.088 | 18.136 | 1.201 | 16.841 | 0.173 | 128.400 | 3.184 | 5.471 | 0.097 |
| **6** | 1.301 | 0.038 | 0.834 | 0.025 | 1.689 | 0.045 | 4.438 | 0.126 | 14.531 | 1.486 | 13.007 | 0.435 | 142.600 | 3.727 | 5.809 | 0.049 |
| LSD | 0.080 |  | 0.056 |  | 0.111 |  | 2.844 |  | 3.757 |  | 3.049 |  | 8.194 |  | 0.226 |  |
| SE(m) | 0.027 |  | 0.019 |  | 0.037 |  | 0.950 |  | 1.255 |  | 1.018 |  | 2.737 |  | 0.076 |  |
| SE(d) | 0.038 |  | 0.027 |  | 0.053 |  | 1.343 |  | 1.775 |  | 1.440 |  | 3.870 |  | 0.107 |  |
| C.V. | 5.856 |  | 5.899 |  | 5.219 |  | 18.253 |  | 8.158 |  | 9.357 |  | 5.415 |  | 2.900 |  |

| **Treatment** | **Fresh flower biomass** | | Dry flower biomass | | **New shoot numbers** | | **K in flower** | | **K uptake in flower** | | **Vase life** | |
| --- | --- | --- | --- | --- | --- | --- | --- | --- | --- | --- | --- | --- |
|  | **Mean** | **S.E.** | **Mean** | **S.E.** | **Mean** | **S.E.** | **Mean** | **S.E.** | **Mean** | **S.E.** | **Mean** | **S.E.** |
| **1** | 16.101 | 0.620 | 1.465 | 0.043 | 0.750 | 0.250 | 0.972 | 0.028 | 10.587 | 3.588 | 6.110 | 0.163 |
| **2** | 18.701 | 1.404 | 1.777 | 0.034 | 1.000 | 0.000 | 0.981 | 0.019 | 17.437 | 0.534 | 6.570 | 0.268 |
| **3** | 20.598 | 0.178 | 1.854 | 0.047 | 1.250 | 0.250 | 1.100 | 0.028 | 25.280 | 4.592 | 6.681 | 0.131 |
| **4** | 23.901 | 1.026 | 1.960 | 0.054 | 1.500 | 0.289 | 1.270 | 0.035 | 37.178 | 6.962 | 7.141 | 0.308 |
| **5** | 26.201 | 1.478 | 2.201 | 0.061 | 1.500 | 0.289 | 1.240 | 0.034 | 46.952 | 5.552 | 7.119 | 0.274 |
| **6** | 31.901 | 0.635 | 2.616 | 0.068 | 2.000 | 0.000 | 1.220 | 0.031 | 63.876 | 2.751 | 8.069 | 0.181 |
| LSD | 3.001 |  | 0.157 |  | 0.660 |  | 0.089 |  | 13.452 |  | 0.689 |  |
| SE(m) | 1.002 |  | 0.052 |  | 0.220 |  | 0.030 |  | 4.493 |  | 0.230 |  |
| SE(d) | 1.417 |  | 0.074 |  | 0.312 |  | 0.042 |  | 6.354 |  | 0.325 |  |
| C.V. | 8.753 |  | 5.297 |  | 33.072 |  | 5.283 |  | 26.780 |  | 6.623 |  |
